# Evaluation of a quality improvement intervention for labour and birth care in Brazilian private hospitals: a protocol

**DOI:** 10.1186/s12978-018-0636-y

**Published:** 2018-11-26

**Authors:** Jacqueline Alves Torres, Maria do Carmo Leal, Rosa Maria Soares Madeira Domingues, Ana Paula Esteves-Pereira, Andreza Rodrigues Nakano, Maysa Luduvice Gomes, Ana Claudia Figueiró, Marcos Nakamura-Pereira, Elaine Fernandes Viellas de Oliveira, Bárbara Vasques da Silva Ayres, Jane Sandall, José M Belizán, Zulmira Hartz

**Affiliations:** 1 Agência Nacional de Saúde Suplementar, Av. Augusto Severo, 84 - Glória, Diretoria de Desenvolvimento Setorial, Rio de Janeiro - RJ, 20021-040 Brazil; 2 Escola Nacional de Saúde Pública Sergio Arouca, Fundação Oswaldo Cruz, Rio de Janeiro, Brazil; 3 Instituto Nacional de Infectologia Evandro Chagas, Fundação Oswaldo Cruz, Rio de Janeiro, Brazil; 4 Casa de Oswaldo Cruz, Fundação Oswaldo Cruz, Rio de Janeiro, Brazil; 5 Faculdade de Enfermagem da Universidade do Estado do Rio de Janeiro, Rio de Janeiro, Brazil; 6 Instituto Nacional de Saúde da Mulher, da Criança e do Adolescente Fernandes Figueira, Fundação Oswaldo Cruz, Rio de Janeiro, Brazil; 7 Department of Women and children’s Health, King’s College London, London, England; 8 Institute for Clinical Effectiveness and Health Policy (IECS), Buenos Aires, Argentina; 9 Instituto de Higiene e Medicina Tropical, Universidade Nova de Lisboa, Lisboa, Portugal

**Keywords:** Caesarean section, Parturition, Maternal and child health, Health evaluation, Implementation research

## Abstract

**Background:**

In Brazilian private hospitals, caesarean section (CS) is almost universal (88%) and is integrated into the model of birth care. A quality improvement intervention, “Adequate Birth” (PPA), based on four driving components (governance, participation of women and families, reorganisation of care, and monitoring), has been implemented to help 23 hospitals reduce their CS rate. This is a protocol designed to evaluate the implementation of PPA and its effectiveness at reducing CS as a primary outcome of birth care.

**Methods:**

Case study of PPA intervention conducted in 2017/2018. We integrated quantitative and qualitative methods into data collection and analysis. For the quantitative stage, we selected a convenient sample of twelve hospitals. In each of these hospitals, we included 400 women. This resulted in a total sample of 4800 women. We used this sample to detect a 2.5% reduction in CS rate. We interviewed managers and puerperal women, and extracted data from hospital records. In the qualitative stage, we evaluated a subsample of eight hospitals by means of systematic observation and semi-structured interviews with managers, health professionals and women. We used specific forms for each of the four PPA driving components. Forms for managers and professionals addressed the decision-making process, implemented strategies, participatory process in strategy design, and healthcare practice. Forms for women and neonatal care addressed socio-economic, demographic and health condition; prenatal and birth care; tour of the hospital before delivery; labour expectation vs. real experience; and satisfaction with care received. We will estimate the degree of implementation of PPA strategies related to two of the four driving components: “participation of women and families” and “reorganisation of care”. We will then assess its effect on CS rate and secondary outcomes for each of the twelve selected hospitals, and for the total sample. To allow for clinical, socio-demographic and obstetric characteristics in women, we will conduct multivariate analysis. Additionally, we will evaluate the influence of internal context variables (the PPA driving components “governance” and “monitoring”) on the degree of implementation of the components “participation of women and families” and “reorganisation of care”, by means of thematic content analysis. This analysis will include both quantitative and qualitative data.

**Discussion:**

The effectiveness of quality improvement interventions that reduce CS rates requires examination. This study will identify strategies that could promote healthier births.

**Electronic supplementary material:**

The online version of this article (10.1186/s12978-018-0636-y) contains supplementary material, which is available to authorized users.

## Plain English summary

There is a global concern about the excess of caesarean section (CS) worldwide. In Brazil, a quality improvement intervention called “Adequate Birth” (PPA) has been implemented to support 23 private hospitals that seek to reduce their CS rates. This study aims to evaluate PPA strategies and their effectiveness at reducing the incidence of CS as a primary outcome of birth care. It will also analyse factors that contributed to the success or failure of PPA implementation. We selected a convenient sample of twelve hospitals. In each hospital, we interviewed and extracted data from hospital records of 400 puerperal women selected at random, in order to detect a 2.5% reduction in CS rate. We also conducted systematic observation and qualitative interviews in a subsample of eight hospitals. The effectiveness of quality improvement interventions at reducing CS rates requires further examination. This study will identify strategies that could promote healthier births.

## Introduction

According to recent estimates, caesarean section (CS) constitutes up to 20% of deliveries worldwide. However, there is a great difference in the use of this procedure across countries and regions. South America has the highest CS rate (42%). This can be attributed in large part to the high rate in Brazil (56%), which has the highest rate among countries studied [1]. It is estimated that 6.2 million cases of CS without clinical indication took place globally in 2008. Even though Brazil only represents 2% of the total number of births in the countries studied, it has contributed to 15% of the total excess of CS, at an estimated cost of US$227 million [2].

Caesarean section is a life-saving intervention. However, evidence from ecological studies shows that, at the population level, caesarean rates above 10–15% are not correlated with further decreases in maternal and neonatal mortality rates [3, 4]. Moreover, cross-sectional and case-control studies conducted in developed and underdeveloped countries have found a correlation between CS and maternal death [5, 6], severe maternal morbidity [7], and maternal near-miss [8, 9]. There are also implications for future pregnancies, since prior uterine scarring might increase the incidence of placenta praevia and accreta [10]. Neonates delivered via CS show lower gut microbiota diversity than those born vaginally [11]. This lower gut microbiota diversity has been associated with long-term adverse outcomes, such as metabolic syndrome [12, 13], type I [14] diabetes, and asthma [15]. Studies have also documented an association between CS and lower breastfeeding rates [16–18]), which may be influenced by factors such as the proportion of caesareans performed without a trial of labour [19], or without consideration of socioeconomic and cultural factors. Elective caesarean rates are very high in Brazil [20].

In Brazil, CS rates in the public and private sectors differ significantly. 80% of all deliveries in the country are carried out in the public sector (healthcare financed by the government), with a CS rate of 43%, whereas in the private sector (healthcare financed by insurance or direct payment) CS is almost universal (88%), constituting 50% of all CS in the country [21]. Policies for the reduction of CS rates in the country must take the characteristics of the private sector into consideration. According to the theoretical-conceptual framework proposed by Torres [22], the model of labour and birth care is closely related to the excess of CS in the private sector. The main features of this model are: (1) medical convenience – time management and the payment model of physicians that lead to CS being advantageous for financial reasons; (2) autonomy-based obstetric practice – link established between the pregnant woman and a sole obstetrician, who takes full responsibility for clinical decision making, independent of network services or other healthcare providers; (3) ‘Maternity hotel’ - private maternity hospitals which focus more on high occupancy rate and aspects of hotel business than on the healthcare role; (4) labour as a purely medical act – the absence of an nurse-midwife or midwife assistant in prenatal and labour care, which is regarded as a purely medical procedure.

Results from national studies have been consistent with the theoretical premises described above. Torres et al. [23] showed that the main factor associated with elective CS in private hospitals in the Southeast region of Brazil was having the same doctor for both prenatal and labour care. Gama et al. [24] demonstrated that nurse-midwives and midwives attended only 16% of vaginal births in Brazil, and that the CS rate was lower in maternity hospitals where these professionals attended labour and birth. Finally, Nakano et al. [25] highlighted that when choosing the maternity hospital for birth, women valued hospitable features more highly, including location, technological facilities and a pleasant atmosphere.

Government action has aimed at changing this scenario. In 2015, after social pressure, the National Agency for Supplementary Health (ANS)—a state body responsible for regulating the Brazilian health insurance market, supported by the Ministry of Health, in partnership with the Institute for Healthcare Improvement (IHI) and the Israelita Albert Einstein Hospital (HIAE)—developed a quality improvement intervention [26] called “Parto Adequado” (“Adequate Birth”) (PPA) [27]. In this type of intervention, through cyclical and incremental implementation of changes, proposed activities are tested and adjusted to the local context, allowing for the implementation and refinement of what works, and discarding what does not work [28, 29]. The PPA is an innovation in the private sector since it represents the first attempt to promote changes in the birth care model with a significant chance of reshaping the determinants of excess CS rates.

The first PPA meeting was in May 2015. The implementation phase of the intervention lasted 18 months and the last meeting of this phase took place in November 2016. PPA strategies are based on international scientific evidence [30] and on two successful instances of CS reduction in Brazilian private hospitals [23, 31].

The four theoretical driving components of PPA are:

1. Governance: forming a coalition between leadership in the health sector, aligning quality and safety in labour and childbirth care;
2. Participation of women and families: empowering women and families so they actively participate in the entire process of pregnancy, birth and postpartum care.
3. Reorganisation of care: reorganising the model of perinatal care to favour the physiological evolution of labour and ensuring that the decision to implement CS is based on clinical criteria;
4. Monitoring: structuring information systems that allow lifelong learning.

**Fig. 1:**
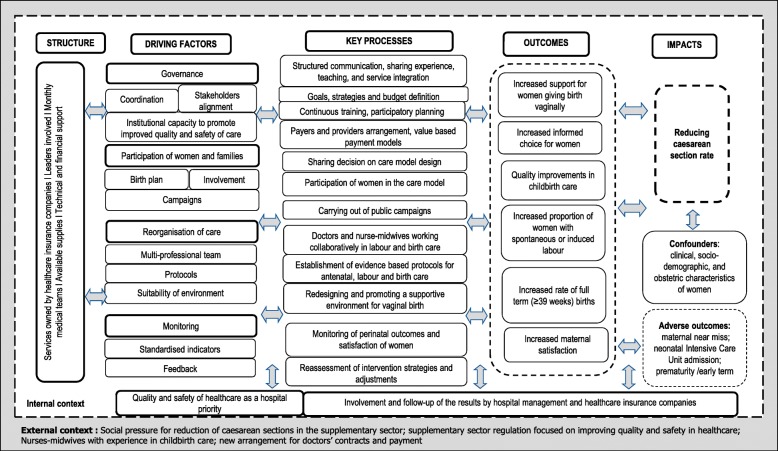
PPA Theoretical Model

The four driving components and the activities related to them are described in the intervention theory (Fig. 1).

The objective of this study is to evaluate the degree of implementation of the intervention (PPA) and its influence on obstetric and perinatal outcomes in twelve private Brazilian hospitals. This paper aims to establish a protocol with regards to the evaluation of the intervention “Adequate Birth” (PPA).

## Method

### Study design

We will conduct hospital-based evaluative research to evaluate the PPA intervention. We will use the “Theory driven evaluation” [32], which is recommended for evaluation of interventions that are not under the control of the evaluator—as is the case for PPA. The “Theory driven evaluation” states that the theory of the intervention must be elucidated in order to evaluate whether the results obtained can be explained by the intervention, or are due to other factors.

The study design is a case study, the case being PPA intervention. We will use mixed methods [33] of research, primarily quantitative, with qualitative components integrated into the data collection and data analysis [34].

We plan to develop both the quantitative and the qualitative components in two phases. In the first phase, we will evaluate the degree of implementation and the effect of the intervention in a sample of hospitals. In the second, we plan to evaluate the degree of implementation and the effect of the intervention in a subsample of hospitals with better performance in the first phase of the evaluative research, 1 year on.

### Quantitative component

#### Criteria for hospital selection

In the first phase of evaluation, we selected a convenient sample of twelve hospitals among the 23 private hospitals included in the PPA intervention. For the selection of these hospitals, we considered three criteria that could have affected the degree of implementation:

- Hospital location according to geographic macro-region (South/Southeast/Midwest and North/Northeast). Brazil is a continental country with relevant social and cultural regional differences. The South/Southeast/Midwest are the richest regions of the country and have the highest CS rates, while the North/Northeast are the poorest, and show the worst health indicators;
- Type of hospital (hospitals owned or not owned by health insurance companies). Hospitals owned by health insurance companies have more autonomy to implement changes as they are independently managed and financed. In private hospitals that do not belong to health insurance companies, funding comes directly from individuals seeking care or those claiming for care on health insurance policies. This may lead to conflicts of interest and obstruct changes;
- Hospital performance: according to administrative data provided by the PPA coordination board, we selected hospitals that reported good and bad results in achieving the PPA CS goals. With these criteria, we expect to evaluate the most and the least successful hospitals with regards to the reduction of CS rates.

**Fig. 2:**
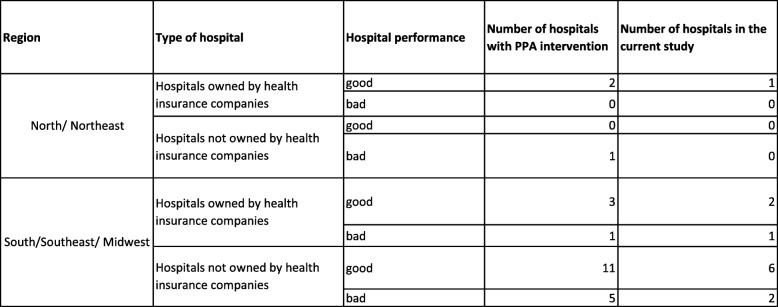
Distribution of hospitals according to selection criteria

The combination of these criteria resulted in eight groups (strata) of hospitals. From the strata with a greater number of hospitals participating in the PPA, we selected a greater number of hospitals. In two strata, there were no hospitals with PPA intervention. In one stratum (North or Northeast regions/hospitals not owned by health insurance company/bad performance) there was only one hospital, which was not included in this evaluative research due to its location and difficulties in interviewer selection and training (Fig. 2).

In the second phase, we plan to select six of the twelve hospitals from the first phase that achieve the most significant reduction in CS rates. Our main objective, when applying these criteria, is to assess the consistency of positive results and to identify the main driving components of these outcomes. In turn, this will contribute to the intervention theory, since it will reveal why the intervention was successful and promote the application of the theory in other contexts that aim to improve the quality of birth care.

### Sample size and post-hoc calculations

Initially, we calculated the sample size, per hospital, necessary for 80% power to detect a reduction of 10% in the number of CS, considering 50% prevalence and a level of significance of 5%. However, in 2014, the year prior to the start of the PPA intervention, the CS varied from 76 to 95% in the twelve hospitals, with a global CS rate of 85%. Considering this, the sample size of 400 women will be 80% accurate at a) detecting a 9% reduction in CS rate in the hospital with the lowest CS rate (76%), and b) detecting a 5% reduction in the hospital with the highest CS rate (95%). Globally, the sample size of 4800 women—12 (hospitals) × 400 (women)—will be 80% accurate at detecting a 2.5% reduction in CS rate. Taking into consideration the first—12 (hospitals) × 400 (women)—and second stages—6 (hospitals) × 400 (women)—, we will include 7200 postpartum women in the study.

### Subjects of the study

All women who were admitted to selected maternity hospitals upon completion of birth of a live newborn (of any gestational age and birth weight) or a stillbirth (with gestational age ≥ 22 weeks and/or birth weight ≥ 500 g).

### Exclusion criteria

All women who gave birth before admission to the hospital; women with extreme communicating difficulty, such as foreigners who could not understand Portuguese; deaf-mute women; women with mental or neurological diseases with severe cognitive impairment; and women who legally interrupted pregnancy.

### Theoretical and practical training of research staff

We developed electronic forms using the application REDCap [35]. Electronic questionnaires enable internal reviews, decreasing the number of typing and filing errors, such as blank or non-applicable spaces, as well as filing invalid numbers (such as dates, age, gestational age, etc.). Moreover, online access to the database allows real-time monitoring of the fieldwork.

Interviewers, supervisors and coordinators participated in theoretical and practical training that lasted 5 days and covered supervisor and interviewer duties, the selection of puerperal women, form filing, electronic data submission using the REDCap platform, ethical considerations, conducting of interviews, and confidentiality of information. We devised instruction manuals for the form filing, which also included the definition of all variables.

We conducted a pilot study at one of the PPA participating maternity hospitals not included in this evaluative research before the fieldwork started. During the pilot study, we tested and adjusted the questionnaires and refined the logistical aspects of the fieldwork.

### Study period

The first phase of data collection took place from March 2017 to August 2017 and the second phase is scheduled to take place from May 2018 to August 2018. The first period of fieldwork began 6 to 8 months after the full implementation of the intervention (PPA). Because of variations in the size of the hospitals, the time required for data collection during the first phase varied from 1 to 4 months, depending on the total number of births per month in each participant hospital. This resulted in a 4 month difference between the first and last hospital in completing data collection.

### Data collection

At the beginning of fieldwork in each hospital, the supervisor in charge interviewed the hospital director or the head of obstetrics or nursing at the obstetric centre. The interview focused on the structure and processes of the hospital (Additional file 1), taking into account the four PPA driving components.

Trained interviewers, 90% of whom were nurse-midwives or midwives, conducted the data collection. They approached all women who were admitted for birth after the beginning of fieldwork and who met the eligibility criteria to participate in the study, until 400 participants were enrolled in each hospital. We included births that took place on weekends and public holidays.

We interviewed women face-to-face—at least 6 h after vaginal births and 12 h after CS births—after they had read and signed the free and informed consent form. This interview included questions on maternal identification; socio-economic condition; previous obstetric history; maternal anthropometric data; prenatal care; illnesses and medication during gestation, labour and birth; and evaluation of care received by woman and newborn (Additional file 2).

We also extracted data from medical records of the women and neonates following their discharge from hospital, including from prenatal cards and ultrasound exams. We collected information regarding prenatal care; hospital admission; labour, birth and infant care; and the use of medication and intervention from these records (Additional file 3). In the case of prolonged hospitalisation, we collected the data on the 28th day of infant hospitalisation, and on the 42nd day of the woman’s hospitalisation. In the case of hospital transfer, we collected data from medical records at the hospital from which the puerperal woman and/or the neonate were discharged.

In a second interview, we contacted women by telephone between 43 and 60 days after birth, a process that took about 3 min, to obtain information on: a) the mother – infections, haemorrhage, re-hospitalisation, and death; b) the infant – respiratory problems, infections, jaundice, rehospitalisation, breastfeeding, and death (Additional file 4). The same team of interviewers who collected data at the hospital conducted the telephone interviews. Puerperal women answered the questions. In the case of either hospitalisation or death, questions were answered by a close relative or companion. All data collected during interviews and from hospital records were related to two PPA driving components “participation of women and families” and “reorganisation of care”.

### Outcomes

The primary outcome was the overall CS rate. The secondary outcomes included: (1) CS rate in accordance with the Robson criteria, a classification system proposed by the World Health Organization (WHO) for assessing, monitoring and comparing CS within and between health facilities [36, 37]. The Robson criteria are based on parity information, onset of labour, gestational age, foetal presentation and number of foetuses, thus forming ten groups of women determined mutually exclusively, inclusively and in terms of clinical relevance [38]; (2) woman’s satisfaction; (3) severe maternal morbidity [39] and maternal near-miss according to the WHO criteria [40]; (4) proportion of preterm births (IG < 37 weeks, determined according to an algorithm developed for the Brazilian population [41]) and early-term births (37 and 38 weeks); and (5) hospitalisation in neonatal intensive care unit, neonatal near-miss and perinatal mortality.

### Missing data

We expect a very low proportion of missing data of variables (< 5%) due to the real-time monitoring of the fieldwork, made possible by online electronic questionnaires for data collection. This strategy enabled internal (by the system) and external (by the supervisors) reviews that decreased the number of typing and filing errors, such as blank or non-applicable spaces, as well as the filing of invalid numbers (such as dates, age, gestational age, etc.). Notwithstanding, missing data will be handled by means of multiple imputation using chained equations. We will apply the Fully Conditional Specification method to obtain five imputed datasets and then adjust our principal models based on these sets using Rubin’s rules to combine effectiveness and standard error estimates.

For women who did not wish to take part in an interview, we asked permission to consult their hospital record. This allowed the data collection of relevant variables.

To account for the loss in follow-up, such as the loss of contacts or refusal of telephone interview, we will apply a logistic regression model. This will estimate the probability that each woman who took part at the baseline (hospital interview) would answer the telephone interview, using a set of variables that differentiate the groups of respondents and non-respondents. Non-response adjustment factors attempt to compensate for the tendency of women of certain characteristics (such as being younger and of lower education) as being less likely to respond, affecting the probability of response in a specific stratum. We will calculate individual sample weights for the analysis of the follow-up interview. The rationale for applying non-response weights is the assumption that non-respondents would have provided similar answers, on average, to respondents from each stratum and adjustment category.

#### Qualitative stage

In the qualitative stage, we evaluated the PPA intervention in a subsample of eight of the twelve hospitals included in the quantitative stage. The inclusion criteria were: regional location, institutional context, and effect on childbirth. We excluded four hospitals because of similarities in geographical location and management model.

The qualitative stage will allow a more comprehensive understanding of how different context factors are linked with the degree of implementation. In addition, this stage should help to explain the influence of success or failure of the implementation of PPA on the CS rate.

The first phase of qualitative research took place from July 2017 to October 2017. Our data collection methods consisted of systematic observation and interviews [42]. The systematic observation plan was based on the following core concepts: organisation of work process; career prospects of health professionals; information register; communication vehicles; regulation and protocol; woman’s care flow; and hospital ambience (Additional file 5). Researchers were immersed in the maternity hospital for 5 days. During that time, the observed data was gathered in field notes as a text file. We used the structure and process questionnaire answered by the hospital manager during the quantitative stage as a reference for the researcher responsible for conducting the systematic observation at the beginning of the activity. This allowed for important issues to be explored during this stage. Analytical treatment will follow the guidelines set out by Bardin’s thematic content analysis [43].

During the same period, we interviewed managers (Additional file 6), obstetric doctors, and nurses (Additional files 7 and 8)*,* whose duties included the implementation of PPA; a total of twelve people in each hospital. Firstly, we invited the director, the PPA leader in the hospital, and the head doctors and nurses of the obstetric centre to engage in the research. However, to investigate alternative explanations or opposing ideas of the project leaders, we also invited obstetric physicians and nurses to participate, since they are directly involved in care for the women and are not in a leadership position. We applied the snowball sampling [44] method. We asked leaders to designate members of the care team who were more committed to the propositions of the project, as well as professionals who were more intolerant to changes. Subsequently, we asked interviewees to nominate colleagues who shared similar opinions regarding PPA, until the sample was exhausted and the data saturated. We held individual interviews using a structured guideline. The interview included questions about the decision-making process of the hospital in opting to participate in PPA; implemented strategies; participation of the healthcare team and the women in strategy design; barriers and facilitators; healthcare practice; monitoring; results; and any other relevant contextual factors related to the intervention. Interviews were face-to-face, digitally recorded and held in reserved rooms in the selected hospitals, with only the interviewee present. Each interview lasted approximately 45 min.

Finally, we intend to interview 50 women in order to learn about their experiences of the PPA, their participation in care flow, and whether their expectation for birth has been fulfilled (Additional file 7). We will select these women according to the information available in the puerperal woman’s questionnaire from the quantitative stage. The sample includes women who sought a natural birth at the beginning of gestation and others who sought CS, as well as women who had the desired outcome and others who did not. Primiparous and multiparous women will form these groups, along with women who were aware of the project and others who were not. We will contact participants via telephone and invite them to participate in this new stage of the research. They will be given the choice of where they wish the interview to be conducted, either in their homes, a room in the maternity hospital, or another place that suits them. The interview will be based on the following thematic topics: prenatal care, selection and visit to the maternity unit, labour expectation versus real experience, impressions of PPA, and access to strategies proposed in the project. Interviews will be digitally recorded and transcribed; to ensure credibility, we will validate the audio and script. At the first stage, we will analyse interviews with both the professionals and the women individually, so that the particular dimensions of the process of implementation in hospitals can be explored. In the following stage, we will compare information from interviews with both groups and from the systematic observation.

### Analysis

**Fig. 3:**
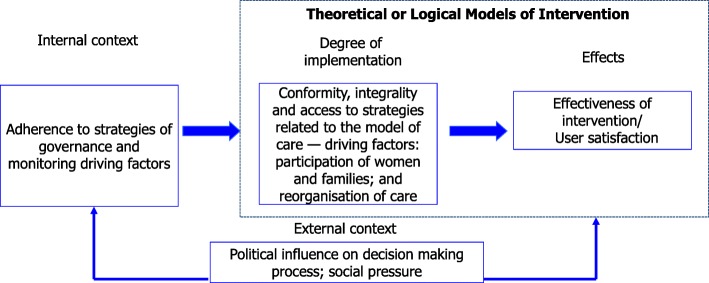
Theoretical evaluation model of the implementation analysis. Legend: Adapted from Hartz et al. 2000. Harz ZMA; Sabroza P; Moreira E & Camacho LAB, 2000. Construção de um modelo para avaliação dos programas de controle de endemias Revista da Sociedade Brasileira de Medicina Tropical, n ° 33 (Suplemento 1): 480–481

We will conduct our analyses based on the evaluation model presented in Fig. 3.

In the first stage, we will estimate the rates and the respective confidence intervals for all outcomes of this study for each hospital and the total sample. We will compare changes in CS rates from the baseline (CS rates available at the Livebirth Information System- Sistema de Informação sobre Nascidos Vivos/SINASC in 2014) to results from the first phase of this evaluative research.

In the second stage, for each hospital and the total sample, we will estimate the degree of implementation of all activities related to the PPA driving components “participation of women and families” and “reorganisation of care” (Table 1). The key activities of both driving components are directly related to the effect of the intervention on the rates of the primary outcome of birth care (CS) [20, 23, 24], (Fig. 3).

**Table 1:** Indicators and source of information to analyse the PPA degree of implementation

Driving components	Indicator (percentage of women)	Data Source
Participation of women and families	Knew that the hospital participated in PPA^a^	Women’s interview (hospital, quantitative)
	Chose the hospital for birth because it participated on PPA intervention^a^	Women’s interview (hospital, quantitative)
	Visited the hospital before birth^a^	Women’s interview (hospital, quantitative)
	Attended antenatal classes at the hospital where she gave birth^a^	Women’s interview (hospital, quantitative)
	Attended antenatal classes at the hospital where she gave birth^a^	Women’s interview (hospital, quantitative)
	Made a birth plan^a^	Women’s interview (hospital, quantitative)
	Had her birth plan respected^a^	Women’s interview (hospital, quantitative)
Reorganisation of care	Had her birth assisted by one of the professionals from the hospital staff^a^	Women’s interview (hospital, quantitative)
	Had her birth assisted by one of the professionals from her antenatal care team ^a^	Women’s interview (hospital, quantitative)
	Had her birth assisted by the same professional who accompanied her throughout antenatal care^b^	Women’s interview (hospital, quantitative)
	Had her vaginal birth assisted by a nurse-midwife^a^	Women’s interview (hospital, quantitative)
	Had a family/friend companionship of her choice during labour^a^	Women’s interview (hospital, quantitative)
	Had a family/friend companionship of her choice during birth^a^	Women’s interview (hospital, quantitative)
	Had to move to another room when it was time to push to give birth^b^	Women’s interview (hospital, quantitative)
	Had a partogram in the hospital medical record^a^	Medical Records (hospital, quantitative)
	Were adimitted in hospital in active phase of labour^a^	Medical Records (hospital, quantitative)
	Had labour induction indicated according to scientific evidence^a^	Medical Records (hospital, quantitative)
	Had freedom to move during labour during labour^a^	Women’s interview (hospital, quantitative)
	Consumed any liquid or food during labor^a^	Women’s interview (hospital, quantitative)
	Used non-pharmacological methods for pain relief^a^	Women’s interview (hospital, quantitative)
	Had a catheter/cannula in her vein during labour^b^	Women’s interview (hospital, quantitative)
	Had an episiotomy^b^	Women’s interview and medical Records (hospital, quantitative)
	Had fundal pressure manouver during birth^b^	Women’s interview (hospital, quantitative)
	Gave birth in litotomy position^b^	Women’s interview and medical Records (hospital, quantitative)

^a^ Judgment criteria of the degree of implementation: 75–100% = satisfactory; 50–74% = partial; < 50% = unsatisfactory

^b^ Judgment criteria of the degree of implementation: < 50% = satisfactory; 50–74% = partial; 75–100% = unsatisfactory

Subsequently, we will estimate the influence of the internal context variables (PPA driving components “governance” and “monitoring”) on the degree of implementation of the activities listed in Table 1. For this analysis, we will use information from the quantitative and qualitative stages (Table 2).

**Table 2:** Dimensions and source of information to analyse the internal context influence on the PPA implementation

Driving components	Dimension	Data Source
Governance	PPA as an estrategic intervention for high hospital management	PPA as an estrategic intervention for high hospital management
	Partnership between hospital and healthcare insurance company to implementing PPA	Interview with hospital mananger (quantitative and qualitative). Interview with health professional from hospital team (qualitative)
	Support and participation of women in PPA desing and implementation	Interview with hospital mananger (quantitative and qualitative). Interview with health professional from hospital team (qualitative). Women interview (qualitative)
	Support and participation of the health professionals in PPA desing and implementation	Interview with hospital mananger (quantitative and qualitative). Interview with health professional from hospital team (qualitative)
	Support from professional societies to hospital implementing PPA	Interview with hospital mananger (quantitative and qualitative). Interview with health professional from hospital team (qualitative)
	Protocols and educational program for doctors and nurse-midwives on evidencebased childbirth care	Interview with hospital mananger (quantitative and qualitative). Interview with health professional from hospital team (qualitative)
	Structured communication process between high hospital management and frontline team to plan and implement PPA strategies	Interview with hospital mananger (quantitative and qualitative). Interview with health professional from hospital team (qualitative); Systematic observation notes
	Favorable ambiance to childbirth care	Interview with hospital mananger (quantitative and qualitative). Interview with health professional from hospital team (qualitative); Systematic observation notes
Monitoring	Structured process to monitore PPA indicators	Interview with hospital mananger (quantitative and qualitative). Interview with health professional from hospital team (qualitative)
	Transparancy of results	Interview with hospital mananger (quantitative and qualitative). Interview with health professional from hospital team (qualitative). Women interview (qualitative); Systematic observation notes

Lastly, we will estimate the effect of the degree of implementation of the activities listed in Table 1 on the CS rate and secondary outcomes. In the univariate analysis, we will use the chi-square method for testing the different proportion of CS according to the degree of implementation with a significance level of 5%. In the multivariate analysis, we will adjust the effect of the degree of implementation on CS rates according to clinical, socio-demographic and obstetric characteristics of the postpartum women for each of the selected hospitals and for the total sample. We will use the following covariates: (1) economic class: A (highest), B, C, D and E (lowest), according to the definition of economic class by the National Research Companies Association – ANEP; (2) education (years of schooling); (3) self-declared skin colour, according to categories used by Instituto Brasileiro de Geografia e Estatística – IBGE in the Demographic Census; (4) anthropometry (pre-gestational weight and height, and the measurements at the end of the pregnancy), either informed or registered in the prenatal card when available; (5) maternal habits: smoking before and during pregnancy and alcohol abuse [45]; (6) obstetric history (parity, presence of uterine scar, prior prematurity, prenatal care, preference for type of birth at the beginning and the end of gestation, complications during pregnancy).

For the six hospitals included in the second phase we will repeat this analysis and will estimate the change in CS rates in the three periods: baseline (2014), and first (2017) and second phase (2018) of the evaluative research. We will use quantitative and qualitative data to try to explain the variation in the degree of implementation and its effect between phase 1 and 2.

## Discussion

The effectiveness of quality improvement interventions at reducing CS rates needs to be assessed, since such evidence-based interventions have the capacity to modify clinical and non-clinical determinants of CS [30]. The development of a complex intervention method involves a systematic approach that should be split into phases [46]. It should begin with pilot studies, proceed to an explanatory evaluation, and subsequently result in an intervention.

It is hoped that this study will enable the identification of strategies to promote healthy births, especially those with a significant effect on: 1) the adoption of good practices during labour and birth; 2) the reduction of unnecessary interventions during labour and birth; 3) the reduction of CS rate; 4) the adoption of scientific evidence based practices in neonatal care; 5) the reduction of adverse neonatal outcomes.

We expect results to be published and used as a guide for implementing changes, thus enabling their promotion and the guidance of hospitals in Brazil and in other countries that are inspired by this work and interested in promoting the enhancement of the labour and birth care model.

The involvement of a wide group of actors for the implementation of PPA, as well as its evaluation, will promote the use of scientific evidence in devising public policies capable of promoting a healthy birth, an essential condition for a full and productive life.

## Additional files

Additional file 1: Hospital Manager Questionnaire. (DOCX 108 kb)

Additional file 2: Postpartum Women Hospital Interview Questionnaire. (DOCX 147 kb)

Additional file 3: Medical Record Questionnaire. (DOCX 130 kb)

Additional file 4: Follow-up Telephone Interview Questionnaire. (DOCX 89 kb)

Additional file 5: Script of Systematic Observation of the Hospital. (DOCX 40 kb)

Additional file 6: Script of Qualitative Interview with Staff. (DOCX 41 kb)

Additional file 7: Script of Qualitative Interview with Health Professionals. (DOCX 42 kb)

Additional file 8: Script of Qualitative Interview with the Mothers. (DOCX 38 kb)

## Abbreviations

ANS: National Agency for Supplementary Health
HIAE: Israelita Albert Einstein Hospital
IHI: Institute for Healthcare Improvement
PPA: Projeto Parto Adequado
SINASC: Sistema de Informação sobre Nascidos Vivos
WHO: World Health Organization
