# Supplementary material for: Evaluation of a quality improvement intervention for labour and birth care in Brazilian private hospitals: a protocol
Source: Reprod Health. 2018 Nov 26;15:194. doi: 10.1186/s12978-018-0636-y (PMC6257968; doi:10.1186/s12978-018-0636-y)
Supplement: Supplementary file 1 — Hospital Manager Questionnaire. (DOCX 108 kb) [file 12978_2018_636_MOESM1_ESM.docx]

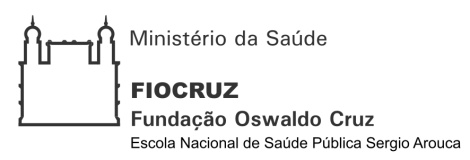


Hospital Manager Questionnaire

Number


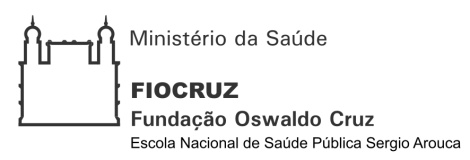


**INFORMATIONS ABOUT STRUCTURE OF MATERNITY HOSPITALS**

NUMBER |___|___|___|

For all questionnaire, fill in **8** or **88** for not applicable and **9** or **99** for not informed.

**I. IDENTIFICATION**

| 1. Date of interview **\|___\|___\|/\|___\|___\|/\|___\|___\|** | 1. Interviewer \|**___\|___\|** | | |
| --- | --- | --- | --- |
| 1. Region: **1.** North **2.** Northeast **3.** Southeast **4.** South **5.** Middle-West | | | \|___\| |
| 1. State **(consultar no instrutivo)** | | | \|___\|___\| |
| 1. City name: ____________________________________________________ | | | |
| 1. Hospital name: ____________________________________________________________________ | | | |
| 1. Hospital number: **(consultar número do estabelecimento no instrutivo)** | | | \|___\|___\|___\| |
| 1. CNPJ \|___\|___\|___\|___\|___\|___\|___\|___\| / \|___\|___\|___\|___\| - \|___\|___\| | | | |
| 1. CNES number \|___\|___\|___\|___\|___\|___\|___\| | | | |
| 1. Address: | | | |
| 1. ZIP code \|___\|___\|___\|___\|___\|-\|___\|___\|___\| | | | |
| 1. Phone number (s):   **\|___\|___\| \|___\|___\|___\|___\|___\|___\|___\|___\|**  **\|___\|___\| \|___\|___\|___\|___\|___\|___\|___\|___\|** | | 1. Fax:   **\|___\|___\| \|___\|___\|___\|___\|___\|___\|___\|___\|** | |

**II. HOSPITAL DESCRIPTION**

| 1. Respondent name: ______________________________________________________________________ | |
| --- | --- |
| 1. Respondent occupation: _________________________________________________________________ | |
| 1. What´s your degree? **(Consider only the main degree)**   **1.** Doctor **2.** Nurse **3.** Other: _____________________________ | \|___\| |
| 1. How is the hospital classified? **(read the alternatives)**   **1.** General or speciality hospital **2.** Only maternity hospital  **3.** Maternal and child hospital **4.** Maternity and gynecology hospital | \|___\|  \|___\| |
| 1. Does the hospital have private beds only? **0.** No, also has public beds **1.** Yes | \|___\| |
| 1. Does the hospital have adult ICU in operation? **0.** No **1.** Yes | \|___\| |
| 1. Does the hospital have intermediary unit in operation? **0.** No **1.** Yes | \|___\| |
| 1. Does the hospital have neonatal ICU in operation? **0.** No **1.** Yes | \|___\| |
| 1. Does the hospital have intermediary neonatal unit in operation? **0.** No **1.** Yes | \|___\| |
| 1. Does the hospital have Kangaroo method beds? **0.** No **1.** Yes | \|___\| |
| 1. Is the hospital a teaching field? **0.** No (**jump to 26**) **1.** Yes | \|___\| |
| 1. Is the teaching field accredited by the Ministry of Education (MEC)?  **0.** No **1.** Yes | \|___\| |
| 1. Does the hospital have any of the following certifications?   **For each option, choose:** **0.** No **1.** Yes **2.** In process of habilitation | |
| 1. Baby-friendly hospital | \|**___\|** |
| 1. Prof. Galba de Araújo prize | \|___\| |
| 1. Others: ___________________________________________________________________ | \|**___\|** |
| 1. Does the hospital have na accreditation program? **0.** No (**jump to 30**) **1.** Yes | \|**___\|** |
| 1. Which one?   1. ONA Organização Nacional de Acreditação  2. JOINT COMISSION International  3.HIMSS – Healthcare Information and Management Systems Society  4.NIAHO – Acreditação Nacional Integrada para Organizações de Saúde  5.Accreditation Canada  6. Other (**answer the 29**) | \|**___\|**  \|**___\|**  \|**___\|** |
| 1. Which one?   ______________________________________________________________________________ |  |
| 1. Does the hospital have acess to blood products? **0.** No **(jump to 32) 1.** Yes | **\|___\|** |
| 1. How is access to blood products?   **1 .** Transfusional agency inside the hospital  **2.** Public supply contract  **3.** Private supply contract | \|**___\|** |
| 1. Where are laboratory tests of hospitalized women and babies performed?   **0.** In the hospital  **1.** In the hospital and in contracted services  **2**. In contracted services | **\|___\|** |
| 1. Where are imaging exams performed on women and babies? **0.** In the hospital   **1.** In the hospital and in contracted services  **2**. In contracted services | **\|___\|** |
| 1. Does the hospital have access to the ambulance to tranfer women in labor? **0.** No   **1.** In the hospital  **2.** In private contracted services | \|___\| |
| 1. Does the hospital have access to the ambulance to transfer of the newborn? **0.** No   **1.** In the hospital  **2.** In private contracted services | \|___\| |

**III. HUMAN RESOURCES**

| 1. With regard to the organization of the medical clinic of obstetrics, this hospital: 2. It is a hospital with open clinical staff 3. It is a hospital owned by Health plan or insurance 4. Both | | | | | | | \|___\| |
| --- | --- | --- | --- | --- | --- | --- | --- |
| With regard to shifts, please inform the number of professionals per **shift in obstetrics (admission + obstetric center)** and their organization: | | | | | | | |
| a.Professionals | b. Number per shift | Forma de Organização dos Plantões | | | | | |
|  |  | c. Professional avaiable 24 hours a day  0.No 1.Yes | d. Professional avaiable in the unit for 12 hours daytime and avaiable at home on night  0.No 1.Yes | e. Profissional avaiable at home for 24 hours  0.No 1.Yes | f. Other | g. (only for who answer *“yes” in options “d, “e” or “f”*)  How long, on average, do the professionals take to get to the establishment when called? | |
| Obstetrician |  |  |  |  |  |  | |
| Neonatologist |  |  |  |  |  |  | |
| Pediatrician |  |  |  |  |  |  | |
| Anesthesiologist |  |  |  |  |  |  | |
| General doctor |  |  |  |  |  |  | |
| Nurse midwife |  |  |  |  |  |  | |
| Midwife |  |  |  |  |  |  | |
| Neonatal nurse |  |  |  |  |  |  | |
| Nurse |  |  |  |  |  |  | |
| Nursing technician |  |  |  |  |  |  | |
| 1. How many births per month, on avarage, are taken care? \|___\|\|___\|\|___\|\|___\| | | | | | | | |
| 1. Approximately what proportion of these deliveries is attended by the hospital's on-call staff? \|___\|\|___\|\|___\|% | | | | | | | |
| **39.** Is there a multidisciplinary team (specialist doctor and / or other professional categories) on stand-by to support in the case of women with complications?  **0**. No (**jump to 41**) **1.** Yes | | | | | | | \|___\| |
| **40.** Which professionals are part of this team?  _______________________________________________________________________________ | | | | | | |  |
| 1. Is there a multidisciplinary team (specialist doctor and / or other professional categories) on stand-by to support not newborns with complications? **0.** No (**jump to 43**) **1.** Yes | | | | | | | \|___\| |
| 1. Which professionals are part of this team?   _______________________________________________________________________________ | | | | | | |  |
| 1. Is there a medical coordinator at the Obstetrics service? **0.** No (**jump to** **46**) **1.** Yes | | | | | | | \|___\| |
| 1. Does this professional have a specialization / residency in Obstetrics? **0.** No **1.** Yes | | | | | | | \|___\| |
| 1. Does this professional play the clinical leadership role with obstetricians who work in the hospital?   **0.** No  **1.** Yes, only for obstetrician hired from the hospital  **2**.Yes, for all obstetricians | | | | | | | \|___\| |
| 1. Is there a medical coordinator at the Neonatology service? **0.** No (**jump to 49**) **1.** Yes | | | | | | | \|___\| |
| 1. Does this professional have a specialization / residency in Neonatology? **0.** No **1.** Yes | | | | | | | \|___\| |
| 1. Does this professional play the clinical leadership role with neonatologists who work in the hospital?   **0.** No  **1.** Yes, only for neonatologists hired from the hospital  **2**. Yes, for all neonatologists | | | | | | |  |
| 1. Is there a professional with a nursing degree who coordinates nursing in the obstetrics service?  **0.** No (**jump to 52**) **1.** Yes | | | | | | | \|___\| |
| 1. Does this professional have a specialization / residency in Obstetrics? **0.** No **1.** Yes | | | | | | | \|___\| |
| 1. Does this professional play the clinical leadership role with nurses who work in the hospital?   **0.** No  **1.** Yes, only for the nurses hired from the hospital  **2.** Yes, for all nurses | | | | | | |  |
| 1. Is there a professional with a nursing degree who coordinates nursing in the Neonatolgy service?   **0.** No (**jump to 55**) **1.** Yes | | | | | | | \|___\| |
| 1. Does this professional have a specialization / residency in Neonatology?   **0.** No **1.** Yes | | | | | | | \|___\| |
| 1. Does this professional play the clinical leadership role with nurses who work in the hospital?   **0.** No  **1.** Yes, only for the nurses hired from the hospital  **2.** Yes, for all nurses | | | | | | |  |
| 1. Professionals from this hospital participated in the following training strategies?   **(For each, choose 0. No or 1. Yes)**  55.a. Open School IHI Course  55.b. Hospital Sofia Feldman workshop for intrapartum care  55.c. Hospital Albert Einstein Course  55.d. PPA Learning Session | | | | | | | \|___\|  \|___\|  \|___\|  \|___\| |

**IV.ORGANIZATION AND INTERNAL STRUCTURING OF WORK**

| 1. Does the hospital have a policy of scheduling cesarean only based on scientific evidence (CEP policy)? **0.** No **1.** Yes | | | \|___\| |
| --- | --- | --- | --- |
| 1. Does the service have standards, clinical protocols and updated written technical routines?   **0.** No (**jump to 59**) **1.** Yes | | | \|___\| |
| 1. Regarding standards, clinical protocols and technical routines, answer: | | |  |
| Content | Is avaiable in the hospital  **0.** No **1.** Yes | How are they avaiable?  1. Session 2. Debate 3. Written 4. Fixed on mural 5. Intranet 6. Others (inform) | |
| Partograph use | \|___\| | \|___\| \|___\| \|___\| \|___\| \|___\| \|___\| | |
| Cardiotocography use | \|___\| | \|___\| \|___\| \|___\| \|___\| \|___\| \|___\| | |
| Anesthesia during labor | \|___\| | \|___\| \|___\| \|___\| \|___\| \|___\| \|___\| | |
| Intrapartum oxytocin | \|___\| | \|___\| \|___\| \|___\| \|___\| \|___\| \|___\| | |
| Cesarean indication | \|___\| | \|___\| \|___\| \|___\| \|___\| \|___\| \|___\| | |
| Antibiotic prophylaxis for cesarean | \|___\| | \|___\| \|___\| \|___\| \|___\| \|___\| \|___\| | |
| Corticosteroids for lung maturation | \|___\| | \|___\| \|___\| \|___\| \|___\| \|___\| \|___\| | |
| Severe preeclampsia and eclampsia management | \|___\| | \|___\| \|___\| \|___\| \|___\| \|___\| \|___\| | |
| Oxytocin for postpartum hemorrhage prophylaxis | \|___\| | \|___\| \|___\| \|___\| \|___\| \|___\| \|___\| | |
| Postpartum hemorrhage management | \|___\| | \|___\| \|___\| \|___\| \|___\| \|___\| \|___\| | |
| Maternal sepsis management | \|___\| | \|___\| \|___\| \|___\| \|___\| \|___\| \|___\| | |
| Criteria for admission in ICU | \|___\| | \|___\| \|___\| \|___\| \|___\| \|___\| \|___\| | |
| GBS sepsis prophylaxis | \|___\| | \|___\| \|___\| \|___\| \|___\| \|___\| \|___\| | |
| Syphilis management during pregnancy | \|___\| | \|___\| \|___\| \|___\| \|___\| \|___\| \|___\| | |
| HIV management during pregnancy | \|___\| | \|___\| \|___\| \|___\| \|___\| \|___\| \|___\| | |
| Newborn immediate care | \|___\| | \|___\| \|___\| \|___\| \|___\| \|___\| \|___\| | |
| Skin-to-skin contact | \|___\| | \|___\| \|___\| \|___\| \|___\| \|___\| \|___\| | |
| Breastfeeding | \|___\| | \|___\| \|___\| \|___\| \|___\| \|___\| \|___\| | |
| Newborn jaundice management | \|___\| | \|___\| \|___\| \|___\| \|___\| \|___\| \|___\| | |
| Newborn infection management | \|___\| | \|___\| \|___\| \|___\| \|___\| \|___\| \|___\| | |
| Newborn hypoglycemia management | \|___\| | \|___\| \|___\| \|___\| \|___\| \|___\| \|___\| | |
| Criteria for admission in NICU | \|___\| | \|___\| \|___\| \|___\| \|___\| \|___\| \|___\| | |
| Others. Detail: |  |  | |
| 1. The hospital has: | | | |
| a. Board Review Committee **0.** No **1.** Yes | | | **\|___\|** |
| b. Death Review Committee **0.** No **1.** Yes | | | **\|___\|** |
| c. Hospital Infection Control Commitee **0.** No **1.** Yes | | | **\|___\|** |
| 1. Does the hospital hold periodic scientific meetings on good practices in the care of labor and delivery? **0.** No **(jump to 62)** **1.** Yes | | | **\|___\|** |
| 1. How often are these scientific meetings held?   1. daily 2. weekly 3. biweekly 4. monthly 5. quarterly 6. semiannual 7. annual  8. without definite term 9. Other. How often? ___________________________________ | | | **\|___\|** |
| 1. Does the hospital routinely monitor process indicators and/or outcomes?   **0.** No (**jump to 67**) **1.** Yes | | | **\|___\|** |
| 1. Is this monitoring done individually for each doctor/nurse who provides care for labor and delivery?   **0.** No **1.** Yes | | | **\|___\|** |
| 1. Which one? **For each item, choose: 0.** No **1.** Yes | | |  |
| 1. Total number of deliveries (vaginal births and cesareans) **(vaginal births including forceps and vaccum)** | | | **\|___\|** |
| 1. Cesarean number/rate | | | **\|___\|** |
| 1. Cesarean number/rate by Robson group | | | **\|___\|** |
| 1. Number of pregnant women with companion during labor and delivery | | | **\|___\|** |
| 1. Number of vaginal births attended by nurses | | | **\|___\|** |
| 1. Number of vaginal births with episiotomy | | | **\|___\|** |
| 1. Number of maternal deaths / maternal mortality ratio | | | **\|___\|** |
| 1. Number of maternal *near miss* cases*/* maternal *near miss* ratio | | | **\|___\|** |
| 1. Number of pregnant and postpartum women admitted in ICU | | | **\|___\|** |
| 1. Number of hysterectomies | | | **\|___\|** |
| 1. Number of women who received blood products | | | **\|___\|** |
| 1. Number of women who were treated with antibiotics | | | **\|___\|** |
| 1. Number of newborns admitted in NICU | | | **\|___\|** |
| 1. Number newborns transferred to referral services | | | **\|___\|** |
| 1. Total number of live newborns | | | **\|___\|** |
| 1. Number/% of low birthweight newborns, below 2500g | | | **\|___\|** |
| 1. Number/% of preterm babies with less than 37 weeks | | | **\|___\|** |
| 1. Number/% of early term babies with 37 – 38 weeks | | | **\|___\|** |
| 1. Number of stillbirths / stillbirth rate | | | **\|___\|** |
| 1. Number neonatal deaths/neonatal mortality rate | | | **\|___\|** |
| 1. Number of early neontal deaths / early neonatal mortality rate | | | **\|___\|** |
| 1. Others: Which ones?___________________________ | | | **\|___\|** |
| 1. Are these indicators disclosed? **0.** No (**jump to 67**) **1.** Yes | | | **\|___\|** |
| 1. In what way? (for each item, choose **0.** No **1.** Yes)   a. Meeting  b. Attached to a mural  c. Intranet  d. hospital website  f. Others. How?: ___________________________________________ | | | **\|___\|**  **\|___\|**  **\|___\|**  **\|___\|** |
| 1. Does the hospital have goals for improving maternal and child care?   **0.** No (**jump to 70**) **1.** Yes | | | **\|___\|** |
| 1. Are these goals shared with the counsil of leaders? **0.** No **(jump to 70)** **1.** Yes | | | **\|___\|** |
| 1. How long does the monthly meeting with the council of leaders address the goals of improving maternal and child care? (*Inform the proportion of the meeting time usually devoted to the discussion of these goals*) | | | **\|__\|\|__\|** |
| 1. Does the hospital have na annual budget to invest in maternal and child care?   **0.** No **1.** Yes | | | **\|___\|** |
| 1. Was the hospital visited by FEBRASGO, ABENFO and the staff of a reference hospital in good practices during labor and delivery? *(****For each item, choose 0. No 1. Yes****)*   70.a. FEBRASGO  70.b. ABENFO  70.c. Reference hospital on good practices. Which one? | | | **\|___\|**  **\|___\|**  **\|___\|** |
| **V. STRUCTURE, EQUIPMENT AND SUPPLIES FOR CARE OF LABOR, DELIVERY, POSTPARTUM AND NEONATAL CARE** | | | |
| 1. Where do pregnant women stay during labor? (can choose more than one, mark the most frequent first)   0. Room/Hospital ward 1. Labor ward/Delivery suite 2. Surgery unit  3. Other. Detail: ________________________________________________________________ | | | \|___\|  \|___\|  \|___\| |
| 1. The place where the pregnant women remains during labor is:   1. Collective 2. Collective but with beds separated by curtains 3. Individual | | | \|___\| |
| 1. Is there an area internal or external for women´s walking (eg porch, solarium, corridors)?   0. No 1. Yes | | | \|___\| |
| 1. Is doula´s companion permitted during hospitalizatiom? 0. No 1. Yes | | | \|___\| |
| 1. Is a companion of woman´s choice allowed?   0. No **(jump to 79)**  1. It´s allowed in specific situations (eg female companion, teenager pregnant woman, woman with some disability, others)  2. It´s allowed for all women | | | \|___\| |
| 1. At what times is the presence of a female companion of the woman's choice allowed during hospitalization?   For each option, choose **0. No or 1. Yes**   1. At admission 2. During labor 3. During vaginal delivery 4. During cesarean 5. During anesthetic recovery 6. After delivery at room 7. After delivery at hospital ward 8. Other. Which? ________________________________________________ | | | \|___\|  \|___\|  \|___\|  \|___\|  \|___\|  \|___\|  \|___\| |
| 1. Is there any additional payment for the woman to have a companion of her choice?   0. No 1. Yes | | | \|___\| |
| 1. Does the hospital have any of the following resources for non-pharmacological pain relief during labor? **For each item, choose: 0.** No **1.** Yes | | | |
| 1. Birthing ball | | | **\|___\|** |
| 1. Rocking birth stool | | | **\|___\|** |
| 1. Ling stairs | | | **\|___\|** |
| 1. Fixed bar | | | **\|___\|** |
| 1. Water in shower | | | **\|___\|** |
| 1. Bath tub | | | **\|___\|** |
| 1. Other: _________________________________________________________ | | | **\|___\|** |
| 1. Does the hospital have a delivery bed that allows delivery in upright positions?   **0.** No **1.** Yes | | | **\|___\|** |
| 1. Does the hospital have a device to control the temperature of vaginal delivery rooms?   **0.** No **1.** Yes, in some rooms **2.** Yes, in all rooms | | | **\|___\|** |
| 1. Does the hospital have a device to control the temperature of cesarean delivery rooms?   **0.** No **1.** Yes, in some rooms **2.** Yes, in all rooms | | | **\|___\|** |
| 1. Does the hospital have a device to control the lighting of vaginal delivery rooms?   **0.** No **1.** Yes, in some rooms **2.** Yes, in all rooms | | | **\|___\|** |
| 1. Does the hospital have a device to control the lighting of vaginal delivery rooms?   **0.** No **1.** Yes, in some rooms **2.** Yes, in all rooms | | | **\|___\|** |
| 1. Where is care given to the newborn after vaginal delivery?   **1.** At the same room where the delivery occurred **2.** At other place | | | **\|___\|** |
| 1. Where is care given to the newborn after cesarean delivery?   **1.** At the same room where the delivery occurred **2.** At other place | | | **\|___\|** |
| **EQUIPMENT FOR MATERNAL EMERGENCY CARE** | | | |
| 1. In situations of emergency and maternal emergency, the childbirth care sector has available **(read the options) For each item, choose: 0.** No **1.** Yes **2.** No, but easy access | | | |
| 1. Mechanical ventilator | | | **\|___\|** |
| 1. Laryngoscope | | | **\|___\|** |
| 1. Orotracheal tube | | | **\|___\|** |
| 1. Bag mask ventilator | | | **\|___\|** |
| 1. Cardiac arrest trolley | | | **\|___\|** |
| 1. Defibrillator | | | **\|___\|** |
| 1. Cardioscope | | | **\|___\|** |
| 1. Pulse oximeter | | | **\|___\|** |
| 1. If there is a need for ICU admission, are there ICU beds in the hospital?   **0.** No (**jump to 90**) **1.** Yes | | | **\|___\|** |
| 1. Are the available ICU beds sufficient for the care of pregnant / puerperal women with complications considering the annual number of hospital deliveries?   0. No 1. Yes | | | **\|___\|** |
| 1. If there is a need to transfer pregnant / postpartum women with complications for admission to an ICU, is there an established reference? 0. No 1. Yes | | | **\|___\|** |
| **EQUIPMENT FOR NEONATAL CARE** | | | |
| 1. What equipment and material exist in the delivery room / procedure area for newborn care? **(read the alternatives)**   **For each item, choose:**  **0.** No **1.** Yes **2.** No, but easy access | | | |
| - 1. Neonatal resuscitation unit | | | \|___\| |
| - 1. Masks | | | \|___\| |
| - 1. Infant stethoscope | | | \|___\| |
| - 1. Neonatal laryngoscope | | | \|___\| |
| - 1. Neonatal orotracheal tube | | | \|___\| |
| - 1. Tracheal cateter | | | \|___\| |
| - 1. Gastric cateter | | | \|___\| |
| - 1. Meconium aspirator device | | | \|___\| |
| - 1. Ventilation material (Mask bag ventilator or portable ventilator with oxygen) | | | \|___\| |
| - 1. Ventilator with manometer and oxygen | | | \|___\| |
| - 1. Baby Puff/ Neo Puff | | | \|___\| |
| 1. *With regard to the Neonatal unit* ***(only for hospitals with Neonatal unit)***   **For each item, choose: 0.** No **1.** Yes | | | |
| 1. What equipment is avaiable to care for newborn: | | | |
| 1. Multi-parameters monitors | | | \|___\| |
| 1. Pulse oximeter | | | \|___\| |
| 1. Mechanical ventilator | | | \|___\| |
| 1. In the NICU is there a chair for mother / father to stay with the newborn?   **0.** No **1.** Yes, for some newborns **2.** Yes, for all newborns | | | \|___\| |
| 1. Does the hospital have a human milk bank? **0.** No **1.** Yes (**jump to 98**) | | | \|___\| |
| 1. Does the hospital have access to pasteurized human milk for newborns admitted to the IU/ICU? **0.** Never **1.** Always **2.** Sometimes | | |  |
| 1. Does the hospital have a place to collect human milk?   **0.** No **1.** Yes | | | \|___\| |
| 1. Does the hospital routinely provide breastfeeding promotion and support actions?   **0.** No **1.** Yes, only during the day **2.** Yes, anytime | | | \|___\| |

***Now I will ask about the hospital pharmacy***

| 1. What is the availability of diferente drugs used for delivery, the puerperium and the newborn in relation to the dispensing of medicines and stock of materials?   **For each, choose**: **0.** Not available **1.** Available **8**. It´s not a routine drug | |
| --- | --- |
| - 1. Beta blockkers (Propranolol, Atenolol, Metoprolol, Pindolol, etc.) | \|___\| |
| - 1. Metildopa (Aldomet, Cardin, Etildopanan, Metildopa) | \|___\| |
| - 1. Hydralazine oral (Apresolina, Nepresol) | \|___\| |
| - 1. Hydralazine injectable (Apresolina, Nepresol) | \|___\| |
| - 1. Nifedipine 10 mg (Adalat, Nifelat, Cardalin, Loncord, Oxcord.) | \|___\| |
| - 1. Alprazolam: Apraz, Frontal, Tranquinal Bromazepam: Brozepax, Lexotam, Nervium, Novazepam, Somalium; Clobazam: Frizium, Urbanil; Clonazepam: Rivotril; Chlordiazepoxide: Psicosedim; Cloxazolam: Elum, Olcadil; Diazepam: Ansilive, Calmociteno, Diazepam, Kiatriun, Noam, Somaplus, Valium; Lorazepam: Lorium, Lorax, Mesmerin) | \|___\| |
| - 1. Corticosteroids (Betamethasone, Celestone soluspan, Dexamethasone) | \|___\| |
| - 1. Methyl ergometrine injectable (Methergin) | \|___\| |
| - 1. Oxytocin (Ocitocina, Orastina, Oxiton, Syntocinon) | \|___\| |
| - 1. Vaginal misoprostol 25 mcg (Citotec, Cytotec ou Misoprostol) | \|___\| |
| - 1. Vaginal misoprostol 200 mcg (Citotec, Cytotec ou Misoprostol) | \|___\| |
| - 1. Tocolytic drugs (Nifedipine, Indometacin, Magnesium sulphate, Atosiban, Terbutaline, Salbutamol e Ritodrine) | \|___\| |
| - 1. Magnesium sulfate | \|___\| |
| - 1. Surfactant (Curosurf, Surfaxin, Exosurf, Survanta) | \|___\| |
| - 1. Antihemorrhagic (except vitamin K) | \|___\| |
| - 1. Vitamin K | \|___\| |
| - 1. Silver nitrate | \|___\| |
| - 1. Rh (D) Immunoglobulin (Rhogam/Matergam/Parthogama) | \|___\| |
| - 1. Penicillin | \|___\| |
| - 1. Metronidazole | \|___\| |
| - 1. Clindamycin | \|___\| |
| - 1. Ampicillin | \|___\| |
| - 1. Cephazolin | \|___\| |
| - 1. Gentamicin | \|___\| |
| - 1. Vancomycin | \|___\| |

**V. CAPACITY INSTALLED**

| 1. We would like to have access to the numbers of operating perinatal beds listed below:   **(Note: Be careful not to repeat beds, that is, if the bed is counted in one item Include again in another, only ACTIVE beds will be counted in places temporarily repaired and deactivated WILL NOT BE ACCOUNTED)** | | |
| --- | --- | --- |
| 1. Total number of maternity operating beds | \|**___**\|**___**\|**___\|** |  |
| 1. Number of Labor Delivery Rooms (LDR) | **\|___**\|**___\|___\|** |  |
| 1. Number of LDR beds | **\|___**\|**___\|___\|** |  |
| 1. Number of predelivery rooms (excluding LDR) | **\|___**\|**___\|___\|** |  |
| 1. Number of predelivery beds (excluding LDR) | **\|___**\|**___\|___\|** |  |
| 1. Number of delivery rooms (excluding LDR) | **\|___**\|**___\|___\|** |  |
| 1. Number of delivery beds (excluding LDR) | **\|___\|___\|___\|** |  |
| 1. Number of obstetric surgery rooms | **\|___**\|**___\|___\|** |  |
| 1. Number of postanesthetic recovery beds | **\|___**\|**___\|___\|** |  |
| 1. Number of obstetric beds with rooming-in | **\|___**\|**___\|___\|** |  |
| 1. Number of obstetric beds without rooming-in | **\|___**\|**___\|___\|** |  |
| 1. Number of obstetric beds for miscarriage/abortion | **\|___**\|**___\|___\|** |  |
| 1. Number of ICU beds | **\|___**\|**___\|___\|** |  |
| 1. Number of intermediary care for women | **\|___**\|**___\|___\|** |  |
| 1. Number of beds for preganant women with complication or high risk | **\|___**\|**___\|___\|** |  |
| 1. Number of beds for mothers whose babies are hospitalized | **\|___**\|**___\|___\|** |  |
| 1. Number of NICU beds | **\|___**\|**___\|___\|** |  |
| 1. Number of neonatal intermediary beds | **\|___**\|**___\|___\|** |  |
| 1. Number of beds for Kangaroo initiave | **\|___**\|**___\|___\|** |  |
| 1. Number of beds in nursery for healthy newborns | **\|___**\|**___\|___\|** |  |
| 1. Number of bed in nursery for newborns with complications | **\|___\|___\|___\|** |  |

**VI. HOSPITAL'S RELATIONSHIP WITH WOMEN USERS**

| 1. Does the hospital provide the pregnant woman's card for women with prenatal care performed at the hospital??   **0.** No  **1.** Yes, regularly **2**. Yes, sporadically 8. Does not perform prenatal care | \|___\| |
| --- | --- |
| 1. Does the hospital offer groups for pregnant women or other educational activities for pregnant women and their families? **0.** No **(jump to 104) 1.** Yes, regularly **2**. Yes, sporadically | \|___\| |
| 1. During participation in pregnant groups, is the woman encouraged to draw up a birth plan?   **0.** No **1.** Yes, regularly **2**. Yes, sporadically |  |
| 1. Does the hospital offer pregnant women and family members access to maternity facilities prior to admission to labor?   **0.** No **(jump to 106) 1.** Yes, regularly **2**. Yes, sporadically | \|___\| |
| 1. Does this visit include the neonatal intensive care unit?   **0.** No **1.** Yes, regularly **2**. Yes, sporadically | \|___\| |
| 1. Does the hospital make any disclosure of the activities developed within the framework of the Adequate Birth Project?   **0**. No **(jump to 108) 1.** Yes, regularly **2**. Yes, sporadically | \|___\| |
| 1. How is this disclosure made? **For each item, choose: 0.** No **1.** Yes 2. Leaflets 3. Hospital website 4. Groups of pregnant women 5. Health insurance company 6. Outhers. Specify: _____________________________________________________ | \|___\|  \|___\|  \|___\|  \|___\|  \|___\| |
| 1. Does the hospital disclose its outcome indicators, such as cesarean rate, mortality rates and other complications, for pregnant users or potential users of the service?   **0.** No **1.** Yes, regularly **2**. Yes, sporadically | \|___\| |
| 1. Does the hospital have ombudsman service? **0.** No **1.** Yes | \|___\| |
| 1. Does the hospital have a patient council?  **0.** No **1.** Yes | \|___\| |
| 1. Does the hospital have a routine mechanism for assessing the satisfaction of women with care received at the institution and obtaining suggestions for improvement?   **0.** No **(jump to 113) 1.** Yes | \|___\| |
| 1. Please describe the work done::   _______________________________________________________________________________________________________________________________________________________________________________________________________________________________________________________________________________________ | |

**VII. ABOUT ADEQUATE CHILDBIRTH PROJECT**

| 1. List the main modifications made to the structure of this hospital after the implementation of the Adequate Childbirth Project.   _______________________________________________________________________________________  _____ __________________________________________________________________________________  _______________________________________________________________________________________  _______________________________________________________________________________________  _________________________________________________________________**______**________________  _______________________________________________________________________________________  _______________________________________________________________________________________  _______________________________________________________________________________________  _____ __________________________________________________________________________________  _______________________________________________________________________________________  _____ __________________________________________________________________________________ | |
| --- | --- |
| 1. List the main changes made in the care process for childbirth and birth in this hospital after the implementation of the Adequate Childbirth Project.   _______________________________________________________________________________________  _____ __________________________________________________________________________________  _______________________________________________________________________________________  _______________________________________________________________________________________  _________________________________________________________________**______**________________  _______________________________________________________________________________________  _______________________________________________________________________________________  _______________________________________________________________________________________  _____ __________________________________________________________________________________  _______________________________________________________________________________________  _____ __________________________________________________________________________________ | |
| 1. In your opinion, what were the main barriers to the implementation of the Adequate Birth project in this hospital??   _______________________________________________________________________________________  _____ __________________________________________________________________________________  _______________________________________________________________________________________  _______________________________________________________________________________________  _________________________________________________________________**______**________________  _______________________________________________________________________________________  _______________________________________________________________________________________  _______________________________________________________________________________________  _____ __________________________________________________________________________________  _______________________________________________________________________________________  _____ __________________________________________________________________________________ | |
| 1. Is there any other question related to this hospital that was not addressed in the questionnaire and which would you like to comment**? 0.** No **(Finalizar a entrevista)** **1.** Yes | **\|___\|** |
| 1. Interviewee's comments:   _______________________________________________________________________________________  _____ __________________________________________________________________________________  _______________________________________________________________________________________  _______________________________________________________________________________________  _________________________________________________________________**______**________________  _______________________________________________________________________________________  _______________________________________________________________________________________  ________________________________________________________________________________________ | |
