# Supplementary material for: Evaluation of a quality improvement intervention for labour and birth care in Brazilian private hospitals: a protocol
Source: Reprod Health. 2018 Nov 26;15:194. doi: 10.1186/s12978-018-0636-y (PMC6257968; doi:10.1186/s12978-018-0636-y)
Supplement: Supplementary file 2 — Postpartum Women Hospital Interview Questionnaire. (DOCX 147 kb) [file 12978_2018_636_MOESM2_ESM.docx]

**
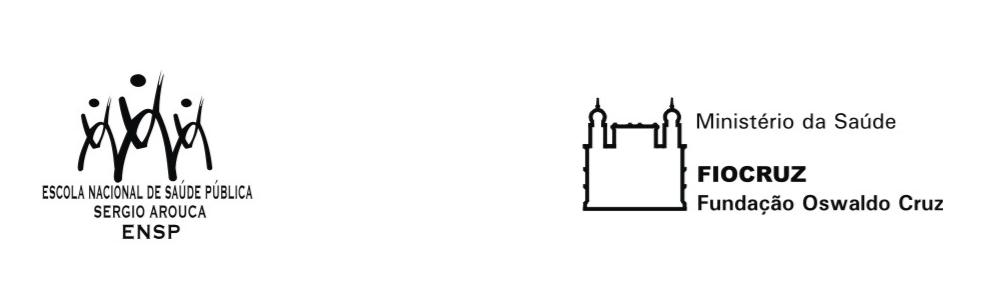
**

**HOSPITAL POSTPARTUM INTERVIEW - DATA COLLECTION INSTRUMENT**

Maternity unit code **|___|___|**

|  |  |
| --- | --- |
| \| 01 \| Hospital Regional Unimed - Fortaleza \| \| --- \| --- \| \| 02 \| Complexo Hospitalar Niterói - Niterói \| \| 03 \| Hospital Pasteur - Rio de Janeiro \| \| 04 \| Vitória Apart - Serra \| \| 05 \| Centro Hospitalar Joinville - Joinville \| \| 06 \| Hospital Moinhos de Vento - Porto Alegre \| \| 07 \| Hospital São Lucas de Santos - Santos \| \| 08 \| Hospital da Luz - São Paulo \| \| 09 \| Hospital Israelita Albert Einstein - São Paulo \| \| 10 \| Hospital Nipo-Brasileiro - São Paulo \| \| 11 \| Hospital Rede D´or São Luiz - Unidade Itaim -São Paulo \| \| 12 \| Hospital Sepaco - São Paulo \| \|  \|  \| |  |
| State: ____________________________________________________________ |  |
| Municipality:_________________________________________________________ |  |
| Name of interviewer: ________________________________________________ |  |
|  |  |
|  |  |
|  |  |
|  |  |

**
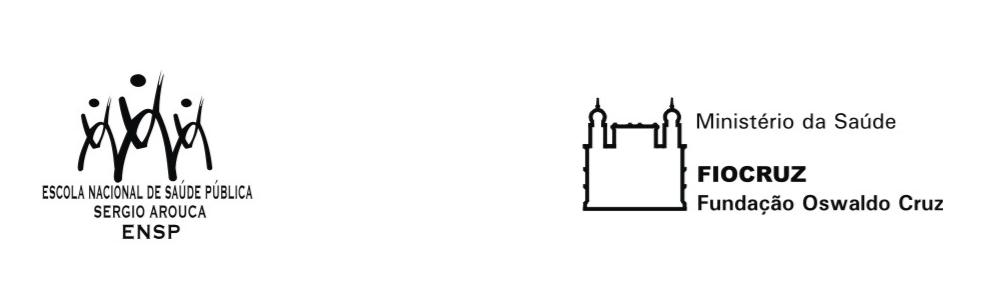
**

RECORD ID |___|___|___|___|___|

**I. GENERAL DATA**

| 1. Time at start of interview___\|___\|:\|___\|___\| | 1. Date of interview\|___\|___\|/\|___\|___\|/\|___\|___\| | |
| --- | --- | --- |
| 1. Date of delivery\|___\|___\|/\|___\|___\|/\|___\|___\| | | |
| 1. Medical records number\|___\|___\|___\|___\|___\|___\|___\|___\|___\|___\|___\|___\|___\|___\|___\| | | |
| 5. Type of pregnancy  **1. Single 2. Twin (two)** | | \|___\| |
| 6. Outcome of newborn  **1. Live 2. Stillbirth 3. Neonatal Death 9. Unknown** | | 1º \|___\|  2º \|___\| |
| 7. Name of newborn  1º ____________________________________________________________  2º ____________________________________________________________ | | |
| 8. Puerperal womans’ full name: ______________________________________________________ | | |
| 9. Puerperal woman mothers’ full name: ________________________________________________ | | |
| - - - 1. Address:   ___________________________________________________________________________________  ___________________________________________________________________________________  ___________________________________________________________________________________ | | |

**II. IDENTIFICATION**

| - - - 1. What is your date of birth? \|___\|___\|/\|___\|___\|/\|___\|___\|\|___\|___\| | | |
| --- | --- | --- |
| - - - 1. How old are you? | \|___\|___\|  (years) | |
| - - - 1. Your skin colour/ ethnicity is ... (Read the alternatives)   **1. White 2. Black 3. Brown/ mulatto/ mixed race 4. Yellow /Asian 5. Indigenous** | | \|___\| |
| - - - 1. Phone numbers (with area code): | | |
| - - - 1. Residential: \|___\|___\| \|___\|___\|___\|___\|___\|___\|___\|___\|___\| (DDD + numbers) | | |
| - - - 1. Mobile: \|___\|___\| \|___\|___\|___\|___\|___\|___\|___\|___\|___\| (DDD + numbers) | | |
| 1. The husband/ partner or a family member's phone number :   \|___\|___\| \|___\|___\|___\|___\|___\|___\|___\|___\|___\| (DDD + numbers) | | |
| 1. The husband/ partner or a family member's name: __________________________________________________ | | |
| 1. Work telephone number (woman's or husband's/partner's) :   \|___\|___\| \|___\|___\|___\|___\|___\|___\|___\|___\|___\| (DDD + numbers) | | |

**III. CLINICAL-OBSTETRIC HISTORY**

| 1. Before the pregnancy with (name of the baby), how many times have you been pregnant, taking into account any abortion or miscarriages you might have had (with less than 5 months’ gestation)?   (**If 00, go to 36)** | | | | \|___\|___\| | |
| --- | --- | --- | --- | --- | --- |
| 1. Before the pregnancy with (name of the baby), have you had any abortion or miscarriage before completing 5 months of pregnancy? How many abortions?   **0. No (go to 23)** | | | | \|___\| | |
| 1. How many abortion/miscarriage were spontaneous? | | | | \|___\|___\| | |
| 1. Before the pregnancy with (name of the baby), how many times have you given birth (to newborns with five or more months’ gestation)?   **(If 00, go to 36)** | | | | \|___\|___\| | |
| 1. How old were you when you had your first delivery (of newborns with 5 or more months’ gestation)? | | | | \|___\|___\| | |
| 1. What was the date of your last delivery (of newborns with five or more months’ gestation) before the current one? | \|___\|___\|/\|___\|___\|/\|___\|___\|\|___\|___\| | | | | |
| 1. How many of these deliveries were vaginal (including forceps and vaccum)? | | | | \|___\|___\| | |
| 1. How many deliveries by caesarean section?   **(if 00, go to 31)** | | | \|___\|___\| | | |
| 1. What was the date of your last caesarean section before the current delivery? | \|___\|___\|/\|___\|___\|/\|___\|___\|___\|___\|\| | | | | |
| 1. What was the reason for the caesarean section before the current pregnancy?   **(Don’t read the options)**   1. Had a previous cesarean section 2. Did not want to feel the pain of a vaginal birth 3. The umbilical cord was around the baby's head 4. Baby was breech/transverse position 5. The baby was large 6. I had no dilatation / the baby did not come down/ the baby did not no fit in the pelvis 7. Post-maturity/post-date 8. The baby was in distress 9. Low amniotic fluid volume 10. High blood pressure 11. Diabetes 12. Another reason not mentioned (answer **30)** | | \|___\|___\|  \|___\|___\|  \|___\|___\|  \|___\|___\|  \|___\|___\|  \|___\|___\|  \|___\|___\|  \|___\|___\| | | | |
| 1. Any other reason not specified above? ___________________________________ | | | | | |
| 1. Before the current pregnancy, have you given birth to any stillborn at five months of gestation or more or weighing more than 500g? | | | | | \|___\|___\| |
| 1. Before the current pregnancy, how many of your children were born alive?   **(If 00, go to 35)** | | | | | \|___\|___\| |
| 1. Before the current pregnancy, have you given birth to a live baby that died within the first month (28 days)? How many? | | | | | \|___\|___\| |
| 1. Before the current pregnancy, have you had a livebirth that died with more than one month (28 days) and less than one year? How many? | | | | | \|___\|___\| |
| 1. Before the current pregnancy, have you given birth to a premature baby (before term)? How many? | | | | | \|___\|___\| |
| 1. Have you ever had a surgery on the uterus (i.e. to remove fibroids, mini-cesarean to interrupt pregnancy, surgery to correct infertility, or to treat uterine problems or other causes?)   **0.No 1.yes** | | | | | \|___\| |

**IV. ANTENATAL CARE**

| 1. When you got pregnant (of the current pregnancy), did you (read options): 2. Wanted the pregnancy at that time 3. Wanted to wait more time to get pregnant 4. Did not want to get pregnant | | | \|___\| |
| --- | --- | --- | --- |
| 1. How did you feel when you found out you were pregnant? (Read options)   1. Satisfied  2. A little satisfied    3. Dissatisfied | | | \|___\| |
| 1. Have you submitted to medical treatment to get pregnant (of the current pregnancy)?   **0. No 1. Yes**  **(If 0, go to 42)** | | | \|___\| |
| 1. Which one?   1. O**vulation drug therapy**  2. Artificial insemination  3. IVF (in vitro fertilization)  4. Other treatment **(specify a 41)** | | | \|___\| |
| 41. Which other treatment? | | | |
| 42. What was the first day of your last menstrual period (before birth)? | \|___\|___\|/\|___\|___\|/\|___\|___\|\|___\|___\| | | |
| 1. Are you certain of this date?   **0. No 1. Yes** | | | \|___\| |
| 1. How many antenatal care visits did you have during the current pregnancy?   ***If she had changed units or attended prenatal care in more than one service, consider the total number of visits.*** | | | \|___\|___\| |
| 1. How many weeks or months of pregnancy were you when you had the first antenatal care visit/first booking? | | 45.1\|___\|___\|weeks  45.2\|___\|months | |
| 1. Where have you had the most antenatal care visits of the current pregnancy?   **(Read options) (only register more than one service if the same number of visits in each)**  **1.** At the same hospital of delivery  **2.** In a private clinic/office  **3.** In the outpatient clinic / health insurance office  **4**. In the public sector  **5.** Others | | | \|___\|  \|___\|  \|___\| |
| 1. What health professional attended you for antenatal care visits during the current pregnancy? (Read options)   1. Doctor 2. Nurse 3. Other 9. Does not know | | | \|___\|  \|___\| |
| During the current pregnancy have you been informed (by yourself or by others) about: **(Read each question)** | | | |
| 48.1 How labour begins?  **0. No 1. Yes** | | | \|___\| |
| 48.2 Danger signs in pregnancy that are alert you to seek the healthcare service?  **0. No 1. Yes** | | | \|___\| |
| 48.3 About things you could do during labour to help the birth (i.e. walking, bathing, birthing positions, non-pharmacological ways to reduce pain, etc.)?  **0. No 1. Yes** | | | \|___\| |
| 48.4 About not cutting the umbilical cord immediately after birth?  **0. No 1. Yes** | | | \|___\| |
| 48.5 About having skin-to-skin contact with the baby in the delivery room?  **0. No 1. Yes** | | | \|___\| |
| 48.6 Breastfeeding within the first hour of birth?  **0. No 1. Yes** | | | \|___\| |
| 1. Where did you get most of this information? 2. At the individual antenatal care consultations 3. At support groups for pregnant women - from antenatal care 4. At support groups for pregnant women - of the hospital you delivered 5. At other support groups for pregnant women 6. From the health insurance plan 7. Internet 8. Others **(specify at 49.1)** | | | \|___\|  \|___\| |
| 49.1 Specify: | | | |
| 1. During antenatal care consultations, were you informed about the risks and benefits of each type of delivery?   **0. No** **1. Yes** | | | \|___\| |

| 1. From what you understood in pregnancy, would you say that in a straightforward pregnancy (without complications): (Read options)   **1**. A vaginal birth is safer for the mother  **2.** A cesarean section is safer for the mother  **3**. Both vaginal birth and cesarean section are safe for the mother  **4.** It was not clear | \|___\| |
| --- | --- |
| 1. Were you considered to have a high-risk pregnancy?   **0. No** **1. Yes** | \|___\| |
| 1. During the current pregnancy were you ever hospitalized?   **0. No** **(go to 54) 1. Yes** | \|___\| |
| 53.1 What was the reason? (**Do not read the options**)   - - 1. Hypertension / preeclampsia     2. Bleeding     3. Threat of preterm birth     4. Urinary tract infection     5. Diabetes     6. Others (**specify at 53.2)** | \|___\|___\|  \|___\|___\|  \|___\|___\|  \|___\|___\| |
| 53.2 Which reason? | |
| 54. Did you know that this hospital participates in a project called Parto Adequado?  0. No **(go to 59)** 1. Yes | \|___\| |
| 55. How did you gert to know?  **1.** Health professional from antenatal care  **2.** Health insurance plan  **3.** Friends  **4.** Midia  **5.** Others **(specify at 55.1)** | \|___\|  \|___\|  \|___\| |
| 55.1 Specify: ________________________________________________________________________ | |
| 1. During the pregnancy with (name of the baby), were you instructed to which hospital / maternity unit to seek care when in labour?   **0. No** **(go to 58) 1. Yes** | \|___\| |
| 1. Who instructed you?   **1**.Health professional from antenatal care  **2**. Health insurance plan  **3.**  Others **(specify at 57.1)** | \|___\|  \|___\| |
| 57.1 Specify: _________________________________________________________________________ | |
| 1. The fact that the hospital participates in *Parto Adequado Project* has influenced your decision to deliver in this hospital?   **0. No** **1. Yes** | \|___\| |
| 1. You were directed to visit this maternity hospital to know the places in which you would be during labor and delivery?   **0. No** **1. Yes** | \|___\| |
| 1. Have you visited the maternity hospital where you would deliver?   **0. No** **1. Yes** **(go to 62)** | \|___\| |
| 1. Why you did not visit the maternity hospital where you would deliver?   **1**. I already knew the maternity  2. Visits were offered during working hours  3. The maternity is far from my home/ work  4. Was not interested in visiting the maternity  **5.** Others **(specify at 61.1)** | \|___\|  \|___\|  \|___\|  \|___\| |
| 61.1 Specify ___________________________________________________________________________ | |
| 1. During this visit : (**read questions 62.1 to 62.4 below**) | |
| 62.1 Were you informed about health situations that would lead to your hospitalization for birth? **0. No** **1. Yes** | \|___\| |
| 62.2 Were you informed about the health team that would assist you at admission for birth?  **0. No** **1. Yes** | \|___\| |
| 62.3 Have you visited the places where you would be during labor and delivery?  **0. No** **1. Yes** | \|___\| |
| 62.4 Have you been informed that you are entitled to a companion without paying for it?  **0. No** **1. Yes** | \|___\| |
| 1. Did the visit help you feel more peaceful about your delivery? **(Ler opções)** 2. Helped a lot 3. Helped a little 4. Did not help or worsen (neutral) 5. Worsened 6. No oppinion | \|___\| |
| 1. Was this maternity where you delivered, the one you visited?   **0. No** **(answer 64.1) 1. Yes** | \|___\| |
| 64.1 Why not? ________________________________________________________________________ | |
| 1. Dis this hospital offer you the possibility to participate in support groups from pregnant women?   **0. No** **(go to 67) 1. Yes** | \|___\| |
| 1. Have you participated in the support group that the hospital offered?   **0. No** **1. Yes** | \|___\| |

**V – DECISION ABOUT TYPE OF BIRTH**

| 1. At the beginning of pregnancy with (name of the baby), what type of birth/mode of delivery did you prefer? 2. Vaginal birth 3. Caesarean delivery 4. Had no preference at all **(go to part VI)** | \|___\| |
| --- | --- |
| 1. In your oppinion what might have influenced your preference, in early pregnancy, in relation to type of birth/mode of delivery? (Do not read the options)   **01.** Stories of births in her family and /or her friends  **02.** Fear of vaginal birth  **03.** She wanted to have a tubal ligation  **04.** The fear of caesarean section  **05.** Previous positive experience with vaginal birth  **06.** Previous positive experience with caesarean  **07.** Previous negative experience with vaginal birth  **08.** Previous negative experience with caesarean  **09.** Vaginal birth is better than cesarean section  **10.** Better recovery in vaginal birth  **11.** To schedule the date for delivery  **12**. Information about the kind of labour  **13.** Other (answer **68.1)** | \|___\|___\|  \|___\|___\|  \|___\|___\|  \|___\|___\|  \|___\|___\|  \|___\|___\|  \|___\|___\|  \|___\|___\|  \|___\|___\|  \|___\|___\|  \|___\|___\| |
| 68.1 In case she reported other reason not included in the options above, describe the reasons here. | |
| 1. During the course of the current pregnancy, has your preference on the type of birth changed?   **0. No** **(go to part VI) 1.** Sim | \|___\| |
| 1. What has influenced this change?   1. Health problems  2. Influence of family or friends  3. Fear of normal birth  4. Fear of cesarean section  5. Information received from the practitioner  6. Information received from other sources  7. Others | \|___\|  \|___\|  \|___\|  \|___\| |
| - 1. Specify others: | |

**VI. ADMISSION TO MATERNITY HOSPITAL**

| 1. When you arrived at the maternity hospital for this hospitalization, were you having painful contractions?   **0. No** **(go to 72) 1. Yes** | \|___\| | |
| --- | --- | --- |
| - 1. At which intervals? **(Read options)**   1. More than one contraction every 5 minutes  2. One contraction every 10 minutes  3. A contraction at intervals greater than 10 minutes  9. Do not remember | \|___\| | |
| 1. How many centimeters dilatated were you at the time of admission? | \|___\|___\| cm | |
| 1. When you arrived at the hospital, were the waters already broken?   **0. No** **1. Yes** | \|___\| | |
| 1. Was the reason for this hospitalization a health complication?   **0. No** **(go to 75) 1. Yes** | \|___\| | |
| - 1. Which one?  1. Hypertension / preeclampsia 2. Diabetes 3. bleeding 4. Placenta praevia / low-lying placenta 5. Infection 6. Problemas related to the fetus 7. Others | \|___\|  \|___\|  \|___\| | |
| 74.2 Specify others? __________________________________________________________________________  _____________________________________________________________________________________ | |  |
| 1. Were you admitted (for the current delivery) for an elective caesaran section?   **0. No** **1. Yes** | \|___\| | |

**VII. LABOUR**

| 1. Did you go into labour and spent part of the time into labour in this hospital where you delivered? **(Read options)** 2. No, because I did not go into labour **(go to 86)** 3. No, because I have arrived with the baby crowning **(go to 86)** 4. No, because I was admitted for an elective caesarean section **(go to 86)** 5. Yes | \|___\| |
| --- | --- |
| 1. What professional assisted your labour? **(Read the options)**    - - 1. Nurse        2. Doctor   **(if only doctor, go to 77.2)** | \|___\|  \|___\| |
| 77.1. The nurse that assisted your labour: (Read the options)  1. Was part of the staff on duty in the hospital  2. Was from the antenatal care team | \|___\|  \|___\| |
| 77.2. The doctor that assisted your labour: **(Read the options)**  1. Was part of the staff on duty in the hospital  2. Was your antenatal care doctor and came to the hospital after being contacted  3. Was from the team of your antenatal care doctor and came to the hospital after being contacted | \|___\|  \|___\| |
| 1. Was your labour induced? That is, did you have a medication inserted into your vagina or a catheter/cannula with a medication (oxytocin) in your vein or a cervical catheter inserted into your vagina to induce the delivery process? 2. No 3. Yes, medication in the vagina 4. Yes, catheter in vein with medication 5. Yes, krause /foley/baloon 6. Do not know | \|___\|  \|___\|  \|___\| |
| 1. Did you consume any liquid or food during your labor? **(Read options)**   **0.** No, I was not offered **1.** No, I did not want **2.** Yes | \|___\| |
| 1. When you were in labour, did you have a catheter/cannula in your vein?   **0. No** **(go to 82) 1. Yes** | \|___\| |
| 1. Was a medication to increase contractions (oxytocin) added? 2. No **1. Yes** **9.** Didn't know | \|___\| |
| This question should only be asked if the waters were at not broken at admission   1. Did anyone break your waters after you arrived at this hospital? (read options) 2. No **(go to 83)** 3. Yes 4. No, it broke by itself during your stay at the hospital **(go to 83)** 5. Didn’t know | \|___\| |
| 82.1 In what moment did they break your waters?   - - - 1. At admission       2. During labour       3. In the delivery room | \|___\| |
| 82.2 How dilated were you (in cm) when they broke your waters? | \|___\| |
| 1. Did you go out of the bed, move around and walk during labour? **(ler as opções)**   **0.** No, because it was not allowed  **1.** No, because you didn’t want to  **2.** Yes | \|___\| |
| 1. Did you do any of the following strategies to relieve pain during labour? (Read options) |  |
| - 1. Bath with warm water   **0.** No, it was nor offered **1.** No, because you didn’t want to **2.** Yes | \|___\| |
| - 1. Shower with warm water   **0.** No, it was nor offered **1.** No, because you didn’t want to **2.** Yes | \|___\| |
| - 1. Birthing ball   **0.** No, it was nor offered **1.** No, because you didn’t want to **2.** Yes | \|___\| |
| - 1. Massage   **0.** No, it was nor offered **1.** No, because you didn’t want to **2.** Yes | \|___\| |
| - 1. Squatting position   **0.** No, it was nor offered **1.** No, because you didn’t want to **2.** Yes | \|___\| |
| - 1. Rocking/birth chair   **0.** No, it was nor offered **1.** No, because you didn’t want to **2.** Yes | \|___\| |
| - 1. Other **(answer 84.8)**   **0. No** **1. Yes** | \|___\| |
| 84.8 If one not specified above, describe here._______________________________________ | |
| 1. Was epidural or spinal anesthesia/analgesia applied on your back anytime during the labour or birth?   **0.** No **1.**Yes **9.** Do not know | \|___\| |
| 1. After admission in this hospital/maternity unit, did you have an exam called CTG (exam with two waist bands around your belly to check contractions and the baby's heart beat)?   **0.** No  **1.** Yes, when you were admitted  **2.** Yes, sometimes during labour  **3.** Yes, throughout all labour  **9.** You don't know | \|___\| |

**VIII. BIRTH**

| 1. With how many weeks of gestation or months of pregnancy was the baby born? | 87.1\|___\|___\| Weeks  87.2\|___\| Months | |
| --- | --- | --- |
| 1. During the current pregnancy did you have an injection (of steroids) to mature the baby's lung? (read options)   0.No **(go to 90) 1**.Yes | \|___\| | |
| 1. How many weeks’ pregnancy were you when you had this injection? | \|___\| \|___\| weeks | |
| 1. Were you encouraged to make a birth plan? 2. No (**go to 91**) 3. Yes | \|___\| | |
| 90.1 Who encouraged you?  **1.** An atenatal care professional  **2.** A team member form the hospital of delivery  **3.** Another person | \|___\|  \|___\|  \|___\| | |
| 1. Did you make a birth plan?   **0. No** (**go to 93**) **1. Yes** | \|___\| | |
| 1. Was your birth plan respected?   **0.** No  **1.** Yes, partialy  **2.** Yes, totally | \|___\| | |
| 1. What professional assisted your delivery? 2. Doctor 3. Nurse | \|___\| | |
| 1. This health care professional who assisted your delivery was:   **(Read the options)**  1. The same professional who accompanied you throughout antenatall care  2. One of the professionals from your antenatal care team  3. One of the professionals from the hospital staff  4. Other | \|___\| | |
| 1. What was the type of birth?   **1.** Vaginal birth/fórceps/ vácuo extrator  **2.** Caesarean section  **(If twins, assign for both deliveries)** | 1º \|___\|  2º \|___\| | |
| 1. Who decided for the type of birth?**(read the options)**   **1.** you  **2.** the doctor  **3.** the nurse  **4.** Joint decision  **5.**Another person **(specify at 96.1)** | \|___\| | |
| - 1. Specify the person here: | | |
| 1. At the time of birth in which position was the baby in your belly? (**Read options**)   1. Vertex (head first position)  2.Breech  3. Other position | | 1º \|___\|  2º \|___\| |

| **ONLY FOR VAGINAL BIRTH**   1. During labour, did you have to move to another room when it was time to push to give birth?   **0.** No **1.**yes | \|___\| |  |
| --- | --- | --- |
| 1. In which position did you have the baby?   1. Lying on your back with legs raised  2. Lying on one side  3. Sitting / reclining  4. in the bathtub  5. All fours support  6. squatting  7. standing up | \|___\| |  |
| 1. At the time of birthing the baby, did someone instruct you to push or put pressure on your belly to help the baby out? (uterine fundal pressure manouvre).   **0. No** **1. Yes** | \|___\| |  |
| 1. At the time of delivery did they perfom an episiotomy (a cut in your perineum/vagina)?   **0.** No **1.**Yes **9.** Didn’t know | \|___\| |  |
| 1. Have you had stitches in your perineum/vagina?   **0.** No **1.**Yes **9.** Didn’t know | \|___\| |  |
| **ONLY FOR CAESAREAN SECTION**   1. At what point it was decided that a caesarean section was necessary?    - - 1. During antenatal care        2. During hospitalization during pregnancy for medical issues        3. At admission to give birth        4. In the labour ward or in the delivery room | \|___\| | |
| 1. What was the reason given for the caesarean section? (**Do not read the options**)   **01.** Wanted a caesarean (fear of vaginal birth, to schedule a date fo delivery, because she thinks it is safer for women and baby and other reasons…)  **02.** Wanted to have a tubal ligation  **03.** The umbilical cord was around the baby's head  **04.** Had a previous caesarean section  **05.** Had two or more previous caesarean sections  **06.** Breech position  **07.** Transverse position  **08.** The baby was big  **09.** The baby had a restricted growth or stopped growing  **10.** Had no dilatation / the baby`s head did not fit or accomodated to the pelvis  **11.** Low amniotic fluid volume / old placenta  **12.** Did not want to feel the pain of vaginal birth  **13.** The baby was in distress  **14.** Post-maturity  **15.** The waters broke  **16.** High blood pressure  **17.** Failed induction / induction did not work  **18.** Another reason not mentioned (answer the question **104.1)** | \|___\|___\|  \|___\|___\|  \|___\|___\|  \|___\|___\|  \|___\|___\|  \|___\|___\|  \|___\|___\|  \|___\|___\|  \|___\|___\|  \|___\|___\|  \|___\|___\|  \|___\|___\|  \|___\|___\|  \|___\|___\|  \|___\|___\|  \|___\|___\|  \|___\|___\| | |
| - 1. If another reason not specified above, describe here: | | |
| 1. Did someone stay with you whilst in hospital/maternity unit stay?   **0. No 1. Yes** **(go to 106)** | \|___\| | |

| - 1. If not, why? **(Do not read the options)**  1. The hospital did not allow any companion  02. No men were allowed as a companion  03. Only allowed companion for teenage mothers  04. Only allowed an adult companion  05. She didn't know she was allowed to have a companion  06. She did not want a companion  07. She didn't have anyone to stay with her  08. Would have to pay to hire a companion  09. The hospital only allowed companion at the delivery room  10. Other (answer the question 105.2   (At the end of this question, go to part **IX)** | \|___\|___\|  \|___\|___\|  \|___\|___\|  \|___\|___\|  \|___\|___\|  \|___\|___\|  \|___\|___\|  \|___\|___\| | |
| --- | --- | --- |
| - 1. If another reason not specified above, describe here: **(go to part IX)** | | |
| 1. Did the person accompaning you stay with you: **(read options below)** | |  |
| - 1. During the hospital admission process (before being admitted)?   0. No 1. Yes 2. No, because you did not want | | **\|___\|** |
| - 1. All the time during labour (before birth)?   0. No 1. Yes 2. No, because you did not want | | **\|___\|** |
| - 1. During birth?   0. No 1. Yes 2. No, because you did not want | | **\|___\|** |
| - 1. During the hospital stay after delivery (stayed with you in the room / ward)?   0. No 1. Yes 2. No, because you did not want | | **\|___\|** |

**IX. NEWBORN INFORMATION – *Not for stillbirths***

| 1. Shortly after giving birth, while in the delivery room, the baby: (**Read options**)   **1.**  Spent some time with you (**go to 109**)  **2.** Went to receive the first care (weighing, measuring, etc)  **3.** Don’t remember (**go to 109**) | | 1º \|___\|  2º \|___\| |
| --- | --- | --- |
| 1. After the first weighing, measuring, etc of the baby, you: (**Read options**) 2. Held the baby 3. Just saw the baby (**go to 110**) 4. Didn`t have any contact (**go to 110**) | | 1º \|___\|  2º \|___\| |
| 1. Did the baby stay in skin-to-skin contact with you, without clothes or sheets between you, shortly after giving birth?   **0. No** **1. Yes** | | 1º \|___\|  2º \|___\| |
| 1. Did the baby came into the postnatal ward with you?   **0. No 1. Yes** **(go to 112)** | | 1º \|___\|  2º \|___\| |
| - 1. Why?   1. The baby was sent to nursery/ warm cradle/ incubator  2. The baby was sent to intermediate or intensive care unit  3. Other reason (answer question 110.2) | | 1º \|___\|  2º \|___\| |
| - 1. Other reason why: | | |
| 1. How long (days, hours or minutes) after birth did your baby come to stay with you in your room? | 1º baby  \|___\|___\| day  \|___\|___\| hours  \|___\|___\| minutes  2º baby  \|___\|___\| day  \|___\|___\| hours  \|___\|___\| minutes | |

**X – BREASTFEEDING**

**(ATTENTION! IN CASE OF STILLBIRTH OR NEONATAL DEATH, DO NOT APPLY THIS PART).**

| 1. Have you offered you breasts to your baby yet?   0. No 1. Yes **(go to 113)** 8. Not applicable | 1º \|___\|  2º \|___\| |
| --- | --- |
| - 1. Why haven`t you offered your breast to your baby yet?   1. Mother is HIV+ (go to part XI)  2. Mother HTLV+ (go to part XI)  3. Premature baby  4. The baby was sick or could not be breastfed  5. The baby has difficulty in sucking  6. Didn`t have enough milk  **7.** I am having trouble in finding a good position to breastfeed  **8.**Others **(specify at 112.2)** | 1º \|___\|  2º \|___\| |
| - 1. Specify: | |
| 1. After birth, did you offer your breasts in the delivery room?   0. No 1. Yes **(go to 115)** | 1º \|___\|  2º \|___\| |
| 1. How long did it take for you to offer your breasts to your baby for the first time? | 1st newborn  \|___\|___\| days  \|___\|___\| hours  \|___\|___\| minutes  2nd newborn  \|___\|___\| days  \|___\|___\| hours  \|___\|___\| minutes |

| 1. (Here) in this hospital, has your baby had any milk or other liquids other than your breast milk?? 2. No **( go to part XI)**   **1.** Yes  **9.** Do not know **( go to part XI)** | 1º \|___\|  2º \|___\| |
| --- | --- |
| - 1. Why did the baby have other milk or liquids? (**Do not read options**)  1. The baby could not suck properly 2. Didn`t have enough milk 3. Routine of the hospital, It was prescribed by the pediatrician 4. Other **(answer 115.2)** | 1º \|___\|\|___\|\|___\|  2º \|___\|\|___\|\|___\| |
| 115.2 describe here:  ______________________________________________________________________________________ | |
| 1. How was the milk / liquid given to your baby? (**Read options**)   1. In the bottle 2. in the cup  3. In the probe / gavage / syringe 4. Other (answer 116.1)  9. Didn `t know the answer | 1º \|___\|\|___\|\|___\|  2º \|___\|\|___\|\|___\| |
| - 1. describe here: | |

**XI. SOCIODEMOGRAPHIC DATA**

| 1. What was the highest level of education you have entered?   **0.** None **(go to 119)**  **1.** Primary or middle school **(answer 118)**  **2.** Secondary/high school **( go to 118)**  **3.** Incomplete University degree **( go to 118)**  **4.**  Complete University degree **(go to 117.2)**  **5.** Postgraduation studies | | \|___\| |
| --- | --- | --- |
| - 1. Which one?      - 1. Lato sensu postgraduation        2. Master degree        3. PhD   (**go to 119**) | | \|___\| |
| 117.2. What is the regulatory duration (in years) of your university course? | | \|___\| |
| 1. What was the last grade you completed in this stage? | | \|___\| |
| 1. What is your marital status? (**Read the options**)   **1.** Single  **2.**  Married / live with partner  **3.**  Separated/ divorced  **4.** Widow | | \|___\| |
| 1. Do you work (and get paid for that) ?   **0.**No **1. Yes** | | \|___\| |
| 1. Who is (the) head of the family?   1.You (go to part XII)  2. partner/husband  3. Your mother 4. Your father 5. Other family member (answer 121.1)  6. Other person that doesn`t live in the same household (answer 121.1) | \|___\| | |
| - 1. If another person not specified above, describe here: | | |
| 1. Which was the highest level of education of the head of the family?   **0.**None **(go to part XII)**  **1.** Primary school **(answer 123)**  **2.** Secondary school **(answer 123)**  **3.** University  9. Didn`t know the answer **(go to part XII)** | | \|___\| |
| 1. What was the last grade the head of the family completed in this stage? ? | | \|___\| |

**XII. HOUSEHOLD INFORMATION**

| 1. The water used in your home comes from? (**Read options**)   1. General distribution network  2. Well or spring  3. Other means | \|___\| | |
| --- | --- | --- |
| 1. Considering the path of the street from your house, you would say that it is :   **1.** Paved / asphalt  **2**. Soil / gravel | \|___\| | |
| 1. How many bathrooms with toilet have in your house? | \|___\|___\| | |
| 1. Now, I will ask you some questions about things you may or may not. | | |
| 1. Do you have microcomputers in your home, considering desktops, laptops, notebooks, and netbooks, and disregarding tablets, palms, or smartphones? If yes how many?   **0. No** | | \|___\| |
| 1. Refrigerator? If yes, how many?   **0. No** | | \|___\| |
| 1. Freezer (independent device or part of refrigerator duplex)? If yes, how many?   **0. No** | | \|___\| |
| 1. DVD? If yes, how many?   **0. No** | | \|___\| |
| 1. Washing machine? If yes, how many?   **0. No** | | \|___\| |
| 1. Do you have a dishwasher in your house? If yes, how many?   **0. No** | | \|___\| |
| 1. Do you have a microwave in your house? If yes, how many?   **0. No** | | \|___\| |
| 1. Do you have clothes dryer in your house? If yes, how many?   **0. No** | | \|___\| |
| 1. Motorcycle? If yes, how many?   **0. No** | | \|___\| |
| 1. Car (for private use)? If yes, how many?   **0. No** | | \|___\| |
| 1. Do you have housemaids in your house? (5 or more days per week)? If yes, how many?   **0. No** | | \|___\| |

**XIII. MATERNAL HABITS**

| 1. Did you smoke before the current pregnancy?   **0. No**  **1. Yes** | \|___\| |
| --- | --- |
| 1. Did you smoke during the current pregnancy?   **0. No** **(go to 143) 1. Yes** | \|___\| |
| 1. Until how many weeks gestation have you smoked during the current pregnancy? | \|___\|___\| |
| 1. Did you use to smoke everyday?   **0. No**  **1. Yes** | \|___\| |
| 142.1 How many cigaretts did you use to smoke per day? | \|___\|___\| |
| 1. During pregnancy, did you drink beer or other alcoholic beverage? 2. No 3. Yes   ***If the woman didn`t drink any alcohol during pregnancy go to part XIV*** | \|___\| |
| 1. Have you ever felt you should Cut down on your drinking? (CAGE & T-ACE)   **0. No** **1. Yes** | \|___\| |
| 1. Does your husband (or parents) ever worry or complain about your drinking? / Have people Annoyed you by criticizing your drinking? (CAGE & T-ACE)   **0. No** **1. Yes** | \|___\| |
| 1. Have you ever had a drink first thing in the morning to steady your nerves or to get rid of a hangover? (Eye opener) (CAGE & T-ACE)   **0. No** **1. Yes** | \|___\| |
| 1. Have you ever awakened in the morning after some drinking the night before and found that you could not remember a part of the evening before?   **0. No** **1. Yes** | \|___\| |
| 1. How many doses do you need to drink to feel "high," that is, how many doses do you need to start feeling different from your "normal" way?   ***( a dose of alcoholic beverage corresponds, for example, to a can or a half bottle of beer, 1 chopp, 2 glasses of beer, 1 glass of wine, a dose of whiskey, cachaça or other distillates or 1 glass of caipirinha)***  **1.** 1 dose  **2**. 2 doses  **3.** 3 doses | \|___\|___\| |

**XIV. CLINICAL HISTORY**

| 1. Did you have some of these diseases before pregnancy was confirmed by a doctor? (Read options below) | |
| --- | --- |
| 1. Heart disease   **0. No** **1. Yes** | \|___\| |
| 1. Non-gestational High blood pressure with prescribed medication for continued use   **0. No** **1. Yes** | \|___\| |
| 1. Severe anemia, not during pregnancy, or other blood disorder   **0. No** **1. Yes** | \|___\| |
| 1. Asthma / bronchitis   **0. No** **1. Yes** | \|___\| |
| 1. Lupus or scleroderma   **0. No** **1. Yes** | \|___\| |
| 1. Hypothyroidism   **0. No** **1. Yes** | \|___\| |
| 155.1. Hyperthyroidism  **0. No** **1. Yes** | \|___\| |
| 1. Non-gestational Diabetes / high blood sugar, confirmed by medical specialist   **0. No** **1. Yes** | \|___\| |
| 1. Kidney disease / kidney confirmed by medical specialist who needs treatment   **0. No** **1. Yes** | \|___\| |
| 1. Epilepsy / seizure before pregnancy   **0. No** **1. Yes** | \|___\| |
| 1. CVA / stroke   **0. No** **1. Yes** | \|___\| |
| 1. Liver disease confirmed by medical specialist who needs treatment   **0. No** **1. Yes** | \|___\| |
| 1. Mental illness, which requires monitoring by a specialist   **0. No** **1. Yes** | \|___\| |
| 1. Other condition 2. No **(go to part XV) 1.**Yes | \|___\| |
| 162.1 Describe other condition__________________________________________________________ | |

**XV– HEALTH INSURANCE**

| 1. Do you have a private medical insurance? (Read options)   **0. No (go to part XVI)**  **1. Yes, one**  **2. Yes, more than one** | \|___\| |
| --- | --- |
| 1. This private medical insurance includes: **(Read options)**   **1.** medical consultations  **2.** hospital admission  **3.** maternity coverage - care during labour and birth  **4.** having health exams | \|___\| \|___\| \|___\| \|___\| |

| 1. Have you used your own resources to have access to any of these procedures? (**Read below)** | |
| --- | --- |
| 165.1 Normal birth or cesarean section scheduled with a doctor from the health insurance? **0. No** **1. Yes** | \|___\| |
| 165.2 Tubal ligation? **0. No** **1. Yes** | \|___\| |
| 165.3 Availability of the professional to accompany your labor?  **0. No** **1. Yes** | \|___\| |
| 165.4 Medical team of your choice?  **0. No** **1. Yes** | \|___\| |
| 165.5 Hospitalization in this maternity hospital? **0. No** **1. Yes** | \|___\| |
| 1. During pregnancy did you request the cesarean rate of doctors and hospitals from the accredited network from your health insurance plan?   **0. No (go to part XVI) 1. Yes** | \|___\| |
| 1. Did the health insurance plan provide the requested information?   **0. No** **1. Yes** | \|___\| |
| 1. This information influenced its decision on: | |
| 168.1 The health professional who assisted your prenatal care?  **0. No** **1. Yes** | \|___\| |
| 168.2 The maternity hospital where you would deliver?  **0. No** **1. Yes** | \|___\| |

**XVI. BIOMETRIC INFORMATION**

| 1. How much did you weigh before pregnancy? (in kg) | | \|___\|___\|___\|,\|___\|Kg |
| --- | --- | --- |
| 1. How much did you weigh at your last antenatal care visit? (in kg) | | \|___\|___\|___\|,\|___\|Kg |
| 170.1 How much did you weigh before delivery? (the closest you know) (in Kg) | | \|___\|___\|___\|,\|___\|Kg |
| 1. When were you last weighed? | \|___\|___\|/\|___\|___\|/\|___\|___\| | |
| 1. How tall are you? (in cm) | | \|___\|___\|___\| cm |

**XVII. SATISFACTION WITH HOSPITAL CARE**

| 1. In the hospital stay for birth, how would you score the clarity in which the health professionals explained things to you?   1.Very good 2. Good 3. Average 4. Poor 5. Very poor | \|___\| |
| --- | --- |
| 1. In the hospital stay for birth, how would you score the respect the professionals had with talking to you and having you at the hospital?   1.Very good 2. Good 3. Average 4. Poor 5. Very poor | \|___\| |
| 1. To receive a respectful treatment also means to have exams performed in a respectful way. In this hospital stay for birth, how do you score the respect the health professionals had with your intimacy during the physical exam and the overall assistance (I.E. during vaginal examination and birth assistance?)   1.Very good 2. Good 3. Average 4. Poor 5. Very poor | \|___\| |
| 1. In the hospital stay for birth, how would you score the time provided to ask questions about your health and your treatment?   1.Very good 2. Good 3. Average 4. Poor 5. Very poor | \|___\| |
| 1. In the hospital stay for birth, how would you score the possibility to discuss with the health professionals about the decisions that were made about the course of you labour and birth?   1.Very likely 2. Likely 3. Not likely or unlikely 4. Unlikely 5. Very unlikely | \|___\| |
| 1. In the hospital stay for birth, do you think you were a victim of maltreatment or any other kind of abuse/ violence by the health professionals, as: (***It is possible more than one answer)***   1.No  2.Verbal abuse (shouted with you or cursed you)  3.Psychological abuse (threatened you, humiliated you or refused to assist you or give you pain relief)  4.Phisical abuse (Push you, hurt you or undertook a painful vaginal examination) | \|___\|  \|___\|  \|___\| |
| 1. In your opinion, the care you received for birth was:   1.Very good 2. Good 3. Average 4. Poor 5. Very poor | \|___\| |
| 1. In your opinion, the care the baby received in the hospital he was born was:   1.Very good 2. Good 3. Average 4. Poor 5. Very poor | \|___\| |

**XIX. FINAL CONSIDERATIONS**

| 1. Would you like to say anything else?   **0. No** **(go to 264) 1. Yes** | \|___\| |
| --- | --- |
| 1. Write down here what she says | |
| 1. Is your prenatal card here with you?   **0. No 1. Yes** | \|___\| |
| 1. Do you have an ultrasound sheet result with you? **0. No 1. Yes** | \|___\| |
| 1. Observations of the interviewer: | |

***Thank you for participating in the interview and remember that we will contact you by phone to ask questions about you and your baby.***
