# Supplementary material for: Evaluation of a quality improvement intervention for labour and birth care in Brazilian private hospitals: a protocol
Source: Reprod Health. 2018 Nov 26;15:194. doi: 10.1186/s12978-018-0636-y (PMC6257968; doi:10.1186/s12978-018-0636-y)
Supplement: Supplementary file 3 — Medical Record Questionnaire. (DOCX 130 kb) [file 12978_2018_636_MOESM3_ESM.docx]

**
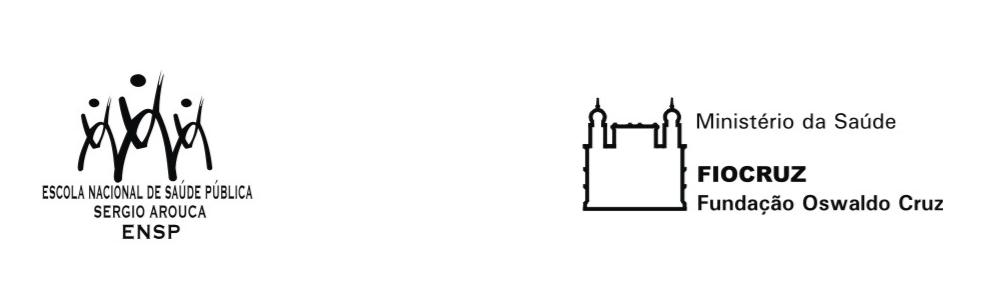
**

**HOSPITAL MEDICAL RECORD - DATA COLLECTION INSTRUMENT**

RECORD ID |___|___|___|___|___|

| **1. GENERAL DATA** | | | | | | | | | | | | | | | | | | | | |
| --- | --- | --- | --- | --- | --- | --- | --- | --- | --- | --- | --- | --- | --- | --- | --- | --- | --- | --- | --- | --- |
| 1. Date of data collection from medical record\|___\|___\|/\|___\|___\|/\|___\|___\| | | | | | | | | | | | | | | | | | | | | |
| 1. Name of post-partum woman_____________________________________________________________ | | | | | | | | | | | | | | | | | | | | |
| 1. Woman`s number in Hospital medical record \|___\|___\|___\|___\|___\|___\|___\|___\|___\|___\|___\|___\|*.* | | | | | | | | | | | | | | | | | | | | |
| 1. Is there any kind of signaling in the medical record concerning the participation of this woman in the “Adequate Birth” project?   0. No 1. Yes | | | | | | | | | | | | | \|___\| | | | | | | | |
| 1. Type of Pregnancy 1. Single 2. Twins (two) | | | | | | | | | | | | | 1º\|___\|  2º \|___\| | | | | | | | |
| 1. Condition at birth   1. Live 2. Stillbirth | | | | | | | | | | | | | \|___\| | | | | | | | |
| **2. Inpatient Information** | | | | | | | | | | | | | | | | | | | | |
| 1. Date of hospital admission: | | | | | \|___\|___\|/\|___\|___\|/\|___\|___\|\|___\|___\| | | | | | | | | | | | | | | | |
| 1. Time of admission: | | | | | \|___\|___\| h \|___\|___\| min | | | | | | | | | | | | | | | |
| 1. Destination from hospital where the woman gave birth:   1. Discharged home/community from hospital  2. Transferred in the postpartum period (go to question 11)  3. Left hospital without medical autorization  4. Death  5. Remained hospitalized after 42 days of birth (go to section 3) | | | | | \|___\| | | | | | | | | | | | | | | | |
| 1. Date of discharge from hospital | | | | | \|___\|___\|/\|___\|___\|/\|___\|___\|\|___\|___\| | | | | | | | | | | | | | | | |
| 1. Hospital where woman was transferred after birth (hospital name - city - state)_______________________________   _________________________________________________________________________________________________ | | | | | | | | | | | | | | | | | | | | |
| **11.1** Reason for being transferred to another hospital:_____________________________________________________ | | | | | | | | | | | | | | | | | | | | |
| **11.2** Destination from hospital where women was transferred to:  1. Discharged home/community from hospital (answer question 11.3 and go to part 3)  2. Left hospital without medical authorization (answer question 11.3 and go to part 3)  3. Death (answer questions 11.3 and 12 and go to part 3)  4. Remained hospitalized after 42 days of birth (go to part 3) | | | | | | | | | | | | | | | | | | \|___\| | | |
| **11.3** Date of discharge from hospital where woman was transferred to | | | | | | | | | \|___\|___\|/\|___\|___\|/\|___\|___\| | | | | | | | | | | | |
| 1. Death Certificate registry number:   \|___\|___\|___\|___\|___\|___\|___\|___\|___\|___\|___\|___\|___\|___\| | | | | | | | | | | | | | | | | | | | | |
| **3. CLINICAL-OBSTETRIC HISTORY** | | | | | | | | | | | | | | | | | | | | |
| **Personal medical history:** | |  | | | | | | | | | | | | | | | | | | |
| 1. Heart disease | | 0. No 1. Yes | | | | | | | | | | | | \|___\| | | | | | | |
| 1. High blood pressure with use of antihypertensive drugs | | 0. No 1. Yes | | | | | | | | | | | | \|___\| | | | | | | |
| 1. Severe anemia or other hemoglobinopathy | | 0. No 1. Yes | | | | | | | | | | | | \|___\| | | | | | | |
| 1. Asthma | | 0. No 1. Yes | | | | | | | | | | | | \|___\| | | | | | | |
| 1. Lupus or scleroderma | | 0. No 1. Yes | | | | | | | | | | | | \|___\| | | | | | | |
| 1. Hypothyroidism | | 0. No 1. Yes | | | | | | | | | | | | \|___\| | | | | | | |
| 18.1. Hyperthyroidism | | 0. No 1. Yes | | | | | | | | | | | | \|___\| | | | | | | |
| 1. Diabetes (non gestacional) | | 0. No 1. Yes | | | | | | | | | | | | \|___\| | | | | | | |
| 1. Chronic kidney disease | | 0. No 1. Yes | | | | | | | | | | | | \|___\| | | | | | | |
| 1. Seizures / epilepsy | | 0. No 1. Yes | | | | | | | | | | | | \|___\| | | | | | | |
| 1. Stroke | | 0. No 1. Yes | | | | | | | | | | | | \|___\| | | | | | | |
| 1. Chronic liver disease | | 0. No 1. Yes | | | | | | | | | | | | \|___\| | | | | | | |
| 1. Psychiatric illness | | 0. No 1. Yes | | | | | | | | | | | | \|___\| | | | | | | |
| 1. Other | | 0. No (go to question 27) 1. Yes | | | | | | | | | | | | \|___\| | | | | | | |
| 1. Specify others______________________________________________________________________________________ | | | | | | | | | | | | | | | | | | | | |
| **Obstetric or medical complications in the current pregnancy (before hospital admission)** | | | | | | | | | | | | | | | | | | | | |
| 1. Cervical incompetence? | | | | | | | 0. No 1. Yes | | | | | | | \|___\| | | | | | | |
| 1. Intra Uterine Growth Restriction (IUGR)? | | | | | | | 0. No 1. Yes | | | | | | | \|___\| | | | | | | |
| 1. Oligohydramnios? | | | | | | | 0. No 1. Yes | | | | | | | \|___\| | | | | | | |
| 1. Polyhydramnios? | | | | | | | 0. No 1. Yes | | | | | | | \|___\| | | | | | | |
| 1. RH alloimmunization? | | | | | | | 0. No 1. Yes | | | | | | | \|___\| | | | | | | |
| 1. Placenta praevia? | | | | | | | 0. No 1. Yes | | | | | | | \|___\| | | | | | | |
| 1. Placenta abruption? | | | | | | | 0. No 1. Yes | | | | | | | \|___\| | | | | | | |
| 1. Premature rupture of membranes? | | | | | | | 0. No 1. Yes | | | | | | | \|___\| | | | | | | |
| 1. Gestational Diabetes? | | | | | | | 0. No 1. Yes | | | | | | | \|___\| | | | | | | |
| 1. Chronic hypertension before pregnancy | | | | | | | 0. No 1. Yes | | | | | | | \|___\| | | | | | | |
| 1. Pre-eclampsia/ gestacional hypertension | | | | | | | 0. No 1. Yes | | | | | | | \|___\| | | | | | | |
| 1. Eclampsia / Seizures? | | | | | | | 0. No 1. Yes | | | | | | | \|___\| | | | | | | |
| 1. HELLP syndrome | | | | | | | 0. No 1. Yes | | | | | | | \|___\| | | | | | | |
| 1. Threat of preterm labour? | | | | | | | 0. No 1. Yes | | | | | | | \|___\| | | | | | | |
| 1. Syphilis? | | | | | | | 0. No 1. Yes | | | | | | | \|___\| | | | | | | |
| 1. Urinary tract infection? | | | | | | | 0. No 1. Yes | | | | | | | \|___\| | | | | | | |
| 1. HIV infection? | | | | | | | 0. No 1. Yes | | | | | | | \|___\| | | | | | | |
| 1. Toxoplasmosis (that needed to be treated)? | | | | | | | 0. No 1. Yes | | | | | | | \|___\| | | | | | | |
| 1. Zika virus infection | | | | | | | 0. No 1. Yes | | | | | | | \|___\| | | | | | | |
| 1. Dengue infection | | | | | | | 0. No 1. Yes | | | | | | | \|___\| | | | | | | |
| 1. Chikungunya infection | | | | | | | 0. No 1. Yes | | | | | | | \|___\| | | | | | | |
| 1. Positive culture for streptococcus in the vagina? | | | | | | | 0. No 1. Yes | | | | | | | \|___\| | | | | | | |
| 1. Newborn with birth defects?   0. No (go to question 51) 1. Yes | | | | | | | | | | | | | | \|___\| | | | | | | |
| 1. Which birth defects? ____________________________________________________________________________ | | | | | | | | | | | | | | | | | | | | |
| 1. Other problems?   0. No (go to question 53) 1. Yes | | | | | | | | | | | | | | \|___\| | | | | | | |
| 1. Which other problems? _________________________________________________________________________ | | | | | | | | | | | | | | | | | | | | |
| 1. Previous uterine surgery (i.e. myomectomy, hysterotomy to interrupt pregnancy, or other uterus surgical procedures)?   0. No 1. Yes | | | | | | | | | | | | | | \|___\| | | | | | | |
| **4. INFORMATION ON HOSPITAL ADMISSION** | | | | | | | | | | | | | | | | | | | | |
| 1. Date of last menstrual period (LMP) | | | | \|___\|___\|/\|___\|___\|/\|___\|___\|\|___\|___\| | | | | | | | | | | | | | | | | |
| 1. Gestational age (on admission) calculated by LMP (in weeks): | | | | \|___\|___\| weeks | | | | | | | | | | | | | | | | |
| 1. Gestational age (on admission) measured by previous ultrasound scan (in weeks): | | | | \|___\|___\| weeks | | | | | | | | | | | | | | | | |
| 1. Gestational age (GA) estimated by the ultrasound scan used for the calculus: | | | | \|___\|___\| weeks | | | | | | | | | | | | | | | | |
| 1. Date of the ultrasound scan used for the GA calculus: | | | | \|___\|___\|/\|___\|___\|/\|___\|___\|\|___\|___\| | | | | | | | | | | | | | | | | |
| 1. Gestational age (on admission) but method of calculation not specified (in weeks): | | | | \|___\|___\| weeks | | | | | | | | | | | | | | | | |
| **60.** Baby`s presentation:  1. Vertex (head first) 2.Breech 3. Other position 9. No registry | | | | | | | | | | 1º newborn \|___\|  2º newborn \|___\| | | | | | | | | | | |
| 1. Any record of blood pressure assessment upon admission?   0. No (go to question 64) 1. Yes | | | | | | | | | | \|___\| | | | | | | | | | | |
| 1. First check of blood pressure: syst (mmHg): | | | | | | | | | | sist \|___\|\|___\|\|___\|mmhg | | | | | | | | | | |
| 1. First check of blood pressure: diast (in mmHg): | | | | | | | | | | diast\|___\|\|___\|\|___\|mmhg | | | | | | | | | | |
| **64.** Is there any record of magnesium sulfate prescription at hospital admission?  0. No 1. Yes | | | | | | | | | | \|___\| | | | | | | | | | | |
| 1. Vaginal bleeding after hospital admission and before delivery?   0. No 1. Yes, small 2. Yes, moderate 3. Yes, intense 4. Yes, unspecified | | | | | | | | | | | | \|___\| | | | | | | | | |
| 1. Loss of amniotic fluid (rupture of membranes) before hospital admission: 2. No 3. Yes, clear liquid 4. Yes, fluid with meconium 5. Yes, bloody fluid 6. Yes, purulent fluid / foul smelling amniotic fluid 7. Yes, unspecified | | | | | | | | | | | | \|___\| | | | | | | | | |
| 1. Dilatation of the cervix on hospital admission in centimeters: | | | | | | | | | | | | \|___\|___\|cm | | | | | | | | |
| 1. Number of contractions every 10 minutes on hospital admission: | | | | | | | | | | | | \|___\|___\| contractions | | | | | | | | |
| 1. Fetal heart rate (FHR) assessment on hospital admission (or the first examination):   0. Absent (go to question 71) 1. Present 9. No registry | | | | | | | | | | | | \|___\| | | | | | | | | |
| 1. Frequency of FHR? | | | | | | | | | | | | \|___\|\|___\|\|___\|bpm | | | | | | | | |
| 1. Any cardiotocography (CTG)? (Allows more than one option)   0. No (go to question 73)  1. Yes, before arrival at the hospital  2. Yes, at the time of hospital admission  3. Yes, during labour | | | | | | | | | | | | \|___\|  \|___\|  \|___\| | | | | | | | | |
| 1. Any abnormality in the CTG? 0. No 1. Yes 9. No registry | | | | | | | | | | | | \|___\| | | | | | | | | |
| 1. Any Fetal Doppler ultrasound? (Allows more than one option)   0. No (go to question 75)  1. Yes, before arrival at the hospital  2. Yes, at the time of hospital admission  3. Yes, during labour | | | | | | | | | | | | \|___\|  \|___\| | | | | | | | | |
| 1. Any abnormality in the fetal Doppler ultrasound?   0. No 1. Yes 9. No registry | | | | | | | | | | | | \|___\| | | | | | | | | |
| 1. Use of corticosteroids before delivery? (Allows more than one option)   0. No (go to question 86) 1. Yes, before arrival at the hospital 2. Yes, at the time of hospital admission | | | | | | | | | | | | \|___\|  \|___\| | | | | | | | | |
| 75.1 Date of corticosteroids use: | | | \|___\|___\|/\|___\|___\|/\|___\|___\|\|___\|___\| | | | | | | | | | | | | | | | | | |
| 1. Diagnosis at the time of hospital admission: (Allows more than one option) 2. Labour prodromes 3. Labour induction 4. Preterm labour/ thread of preterm labour 5. Active Labour 6. Ruptured membranes 7. Multiple pregnancy (two fetuses or more) 8. Prolonged pregnancy / post-maturity 9. Fetal distress (acute / chronic) 10. Abnormal Fetal Doppler ultrasound (umbilical or cerebral) 11. Intra uterine growth restriction (IUGR) 12. Polyhydramnios 13. Oligohydramnios 14. Placental abruption 15. Vaginal bleeding 16. Placenta praevia 17. Chronic hypertension 18. Pre-eclampsia 19. Eclampsia / seizures 20. HELLP syndrome 21. Breech presentation 22. other abnormal presentation ( transverse) 23. Previous c-section 24. Non gestational diabetes 25. Gestational Diabetes 26. HIV Infection 27. Fetal death 28. Sepsis 29. Elective caesarean section 30. No clinical or obstetric diagnosis registred 31. Other diagnosis **(**answer question 77) 32. Clinical complications (go to question 78) | | | | | | | | | | | | | | | | | \|___\|  \|___\|  \|___\|  \|___\|  \|___\|  \|___\|  \|___\|  \|___\|  \|___\|  \|___\|  \|___\|  \|___\|  \|___\|  \|___\|  \|___\|  \|___\|  \|___\|  \|___\|  \|___\|  \|___\|  \|___\|  \|___\|  \|___\|  \|___\|  \|___\|  \|___\|  \|___\|  \|___\| | | | |
| 1. Which other diagnosis? _________________________________________________________________________ | | | | | | | | | | | | | | | | | | | | |
| 1. Which medical complications? ______________________________________________________________________ | | | | | | | | | | | | | | | | | | | | |
| **5. LABOUR CARE** | | | | | | | | | | | | | | | | | | | | |
| 1. Type of Labour 2. Spontaneous labour 3. Inducted labour 4. Failed induction 5. No labour (answer the question 80 and go to question 102) | | | | | | | | | | | | | | | \|___\| | | | | | |
| 1. Was there a caesarean section indication at the time of hospital admission?   **0.** No  **1.** Yes  *IF “Yes” and “spontaneous labour” at question 79,* ***go to question 102****.*  *If “yes” and “Inducted labour” or “Failed induction” at question 79* ***answer question 83 and go to question 102*** | | | | | | | | | | | | | | | | | | | | \|___\| |
| 1. Date of admission at the labour ward: | | | | | | \|___\|___\|/\|___\|___\|/\|___\|___\|\|___\|___\| | | | | | | | | | | | | | | |
| 1. Time of admission at the labour ward: | | | | | | | | | | | \|___\|___\| h \|___\|___\| min | | | | | | | | | |
| *This question must be answerd whenever the answer to question 79 is “Inducted labour” or “Failed Trial of labour”*   1. Medications/ methods used for labour induction   **1.** Oxytocin  **2.**Misoprostol  **3.**Krause /foley/baloon  **4.** Dinoprostone | | | | | | | | | | | \|___\|  \|___\| | | | | | | | | | |
| 1. Diet prescription during labour:   0. None 1. Liquid diet 2. Another type of diet 9. No registry | | | | | | | | | | | \|___\| | | | | | | | | | |
| 1. Intravenous fluids prescription during labour:   0. No 1. Yes (go to question 87) | | | | | | | | | | | \|___\| | | | | | | | | | |
| 1. Placement of peripheral venous access during labour:   0. No 1. Yes | | | | | | | | | | | \|___\| | | | | | | | | | |
| 1. Antibiotic prescription during labour: 0. No 1. Yes | | | | | | | | | | | \|___\| | | | | | | | | | |
| 1. Shaving for birth (at the hospital)? 0. No 1. Yes | | | | | | | | | | | \|___\| | | | | | | | | | |
| 1. Enema before delivery? 0. No 1. Yes | | | | | | | | | | | \|___\| | | | | | | | | | |
| 1. Care provider during labour: (Allows more than one option)   1. Medical doctor  2. Obstetric nurse  3. Nurse  4.Other  9. No registry | | | | | | | | | | | \|___\|  \|___\|  \|___\|  \|___\| | | | | | | | | | |
| 1. Was there a partogram in the hospital medical record:   0. No (go to question 94) 1. Yes | | | | | | | | | | | \|___\| | | | | | | | | | |
| 1. Cervical dilatation at the partograph first registration? | | | | | | | | | | | \|___\|\|___\|cm | | | | | | | | | |
| 1. How many times during labour were there clinical and obstetric information recorded at the partograph? | | | | | | | | | | | \|___\|\|___\| | | | | | | | | | |
| 1. Synthetic oxytocin prescription during labour?   0.No (go to 96) 1. Yes | | | | | | | | | | | \|___\| | | | | | | | | | |
| 1. Uterus cervix dilatation when oxytocin was administered (in centimeters) | | | | | | | | | | | | | | | | \|___\|\|___\|cm | | | | |
| 1. Pain relief drugs prescription during labour (Allows more than one option)   1. No 2. Yes, opioids (Mepergan, meperidine, demerol or pethidine) 3. Yes, other (buscopam, dipyrone, hyoscine) | | | | | | | | | | | | | | | | \|___\|  \|___\| | | | | |
| 1. Use of anesthesia/analgesia during labour:   0. No 1. Epidural 2. Spinal 3. Spinal + epidural (combined) 4. General | | | | | | | | | | | | | | | \|___\| | | | | | |
| 1. Rupture of membranes during labour/delivery: (If membranes ruptured before hospital admission, register “8”)   1. Yes, spontaneous rupture  2. Yes, artificial rupture  3. Yes, unspecified | | | | | | | | | | | | | | | \|___\| | | | | | |
| 1. Uterus cervix dilatation at the time of rupture of membranes (in cm): | | | | | | | | | | | | | | | \|___\|\|___\|cm | | | | | |
| 1. Characteristic of amniotic liquid::   **1.** Clear liquid  2. Fluid with meconium  3. Bloody fluid  4. Purulent fluid / foul smelling amniotic fluid  5. Unspecified | | | | | | | | | | | | | | | \|___\| | | | | | |
| 1. Was there any of the following conditions registered at the hospital medical chart? (Allows more than one option ) 2. Fetal distress during labour 3. Elimination of thick meconium 4. Fetal bradycardia (BCF <110) 5. Fetal tachycardia (BCF> 160) 6. Presence of late decelerations (slowdown in cardiotocography) 7. CTG **-** categories 2 or 3 8. Diagnosis of chorioamnionitis. or intrauterine infection before birth 9. No record of any of the above conditions | | | | | | | | | | | | | | | | \|___\|  \|___\|  \|___\|  \|___\|  \|___\| | | | | |
| **6. BIRTH CARE** | | | | | | | | | | | | | | | | | | | | |
| 1. Date of birth? | | | | \|___\|___\|/\|___\|___\|/\|___\|___\|\|___\|___\| | | | | | | | | | | | | | | | | |
| 1. Time of birth? | | | | | | | | \|___\|___\| horas\|___\|___\| min | | | | | | | | | | | | |
| 1. Type of delivery?   1. Vaginal (including forceps/vacuum)  2. Cesarean (go to question 114) (In case of twins with both vaginal and cesarean birth, complete with both types of birth) | | | | | | | | | | | | | | | | | \|___\|  \|___\| | | | |
| 1. Use of forceps / vacuum extractor?   0. No 1. Forceps 2. Vacuum | | | | | | | | | | | | | | | | | \|___\|  \|___\| | | | |
| 1. Care provider during birth?   **1.** Medical doctor  **2.** Obstetric nurse  **3.** Nurse  **4.**Other  **9.** No registry | | | | | | | | | | | | | | | | | \|___\| | | | |
| 1. Last cervical dilatation registered at partograph/hospital chart before birth | | | | | | | | | | | | | | | | | \|___\|\|___\|cm | | | |
| - 1. Time when the pregnant woman reached full dilatation (in partogram or medical records): | | | | | | | | | | \|___\|___\| h\|___\|___\| min | | | | | | | | | | |
| 1. Episiotomy:   0. No 1. Yes | | | | | | | | | | | | | | | | | \|___\| | | | |
| 1. Occurrence of vaginal/perineal lacerations?   0. No  1. Yes, first degree 2. Yes, second degree  3. Yes, third degree  4. Yes, fourth degree  5. Yes, unkown degree | | | | | | | | | | | | | | | | | \|___\| | | | |
| 1. Ocurrance of vaginal/perineal suture or episiotomy suture?   0. No  1. Yes  9. No registry | | | | | | | | | | | | | | | | | \|___\| | | | |
| 1. Regional anesthesia before suture or episiotomy suture?   0. No 1. Yes 9. No registry | | | | | | | | | | | | | | | | | \|___\| | | | |
| 1. Shoulder dystocia? 0. No 1. Yes | | | | | | | | | | | | | | | | | 1º newborn \|___\|  2º newborn \|___\| | | | |
| 1. Umbilical Cord prolapse? 0. No 1. Yes | | | | | | | | | | | | | | | | | 1º newborn \|___\|  2º newborn \|___\| | | | |
| **7. C section indication** | | | | | | | | | | | | | | | | | | | | |
| 1. Obstetrician Information: (check the surgery report and register the indications following the same order described by the assistant obstetrician) | | | | | | | | | | | | | | | | | | | | |
| 1. Mother request 2. Previous uterine scar other than c-section 3. Previous Cesarean Section 4. Tubal ligation 5. Umbilical Cord 6. Breech presentation 7. Transverse presentation 8. Cephalopelvic Disproportion (CPD) 9. Failure to Progress 10. Placenta praevia 11. Placenta abruption 12. Fetal distress 13. Intrauterine Growth Restriction (IUGR) 14. Fetal Doppler ultrasound abnormalities 15. Ruptured membranes 16. Hypertension / Pre-eclampsia 17. Eclampsia 18. HELLP syndrome 19. Diabetes 20. Oligohydramnios 21. Polidramnia 22. Twin pregnancy 23. Preterm 24. GA > 41 weekss/ Postmaturity (Prolonged Pregnancy) 25. Macrosomia 26. Failed induction 27. Malformation 28. Fetal death 29. HIV Infection 30. Clinical complications 31. Not specified in the medical records 32. Others(answer question **116)** | | | | | | | | | | 1ª information \|___\|\|___\|  2ª information \|___\|\|___\|  3ª information \|___\|\|___\|  4ª information \|___\|\|___\| | | | | | | | | | | |
| 1. Another. Which?_______________________________________________________________________________ | | | | | | | | | | | | | | | | | | | | |
| 1. type of Anaesthesia: 1. Epidural   2. Spinal  3. Spinal + epidural  4. General | | | | | | | | | | | | | | | | | \|___\| | | | |
| 116.1 Prophylactic antibiotics prescription/use:  0. No  1. Yes  9. No registry | | | | | | | | | | | | | | | | | \|___\| | | | |
| 117. Perioperative complications  0. No  1. Bladder injury  2. Ureter injury  3. Bowel injury  4.Other types of lesion (answer question 118) | | | | | | | | | | | | | | | | |  | | | |
| 1. Which lesions?__________________________________________________________________________ | | | | | | | | | | | | | | | | | | | | |
| **8. Immediate puerperium** | | | | | | | | | | | | | | | | | | | | |
| 1. Prescription / use of anti-human immunoglobulin for RH incompatibility   0. No 1. Yes | | | | | | | | | | | | | | | | | \|___\| | | | |
| 1. Prescription / use of prophylactic oxytocin after immediate delivery?   0. No 1. Yes | | | | | | | | | | | | | | | | | \|___\| | | | |
| 1. Was there postpartum haemorrhage?   0. No (go to question 123) 1. Sim | | | | | | | | | | | | | | | | | \|___\| | | | |
| 1. Was there severe postpartum haemorrhage?   0. No 1. Yes | | | | | | | | | | | | | | | | | \|___\| | | | |
| 1. Was there a diagnosis of uterine atony / hypotonia?   0. No 1. Yes | | | | | | | | | | | | | | | | | \|___\| | | | |
| 1. Prescription / use of any treatment with uterotonic? (Allows more than one option)   0. No  1. Oxytocin  2. Methergin  3. Misoprsotol | | | | | | | | | | | | | | | | | \|___\|  \|___\|  \|___\|  \|___\| | | | |
| 1. Was there a diagnosis of retained placenta? 0. No 1. Yes | | | | | | | | | | | | | | | | | \|___\| | | | |
| 1. Was there a diagnosis of placenta accreta? 0. No 1. Yes | | | | | | | | | | | | | | | | | \|___\| | | | |
| 1. Uterine rupture? 0. No 1. Yes | | | | | | | | | | | | | | | | | \|___\| | | | |
| 1. Blood transfusion? 0. No (go to question 130)   1. Yes, before delivery  2. Yes, after delivery | | | | | | | | | | | | | | | | | \|___\| | | | |
| 1. How many units of blood? | | | | | | | | | | | | | | | | | \|___\|\|___\| | | | |
| 130. Interventional radiology for treatment of postpartum haemorrhage?  0. No 1. Yes | | | | | | | | | | | | | | | | | \|___\| | | | |
| 1. Was there a diagnosis of postpartum endometritis? 0. No 1. Yes | | | | | | | | | | | | | | | | | \|___\| | | | |
| 1. Surgical or perineal wound infection? 0. No 1. Yes | | | | | | | | | | | | | | | | | \|___\| | | | |
| 1. Sepsis or severe systemic infection?   **0.** No **1.** Yes, before delivery  **2.** Yes, after delivery | | | | | | | | | | | | | | | | | \|___\| | | | |
| 1. Prescription / use of antibiotics? (In the case of cesarean section, do not consider prophylactic antibiotic)   **0.** No (go to question 137) 1. Yes | | | | | | | | | | | | | | | | | \|___\| | | | |
| 1. How many days of antibiotic use? | | | | | | | | | | | | | | | | | \|___\|\|___\| | | | |
| 1. Which antibiotics? ___________________________________________________________________________ | | | | | | | | | | | | | | | | | | | |  |
| 1. Return to the operating room (curettage, extraction of placenta, drainage of hematomas, abdominal bleeding)?   0. No 1. Yes | | | | | | | | | | | | | | | | | \|___\| | | | |
| 1. Postpartum Laparotomy?   0. No 1. Yes | | | | | | | | | | | | | | | | | \|___\| | | | |
| 1. Severe postpartum hypertension?   0. No 1. Yes | | | | | | | | | | | | | | | | | \|___\| | | | |
| 1. Severe Pre-eclampsia?   0. No 1. Yes, before delivery 2. Yes, after delivery | | | | | | | | | | | | | | | | | \|___\| | | | |
| 1. Eclampsia?   0. No 1. Yes, before delivery 2. Yes, after delivery | | | | | | | | | | | | | | | | | \|___\| | | | |
| 1. HELLP syndrome?   0. No 1. Yes | | | | | | | | | | | | | | | | | \|___\| | | | |
| 1. Prescription / use of magnesium sulphate?   0. No  1. Yes, before delivery  2. Yes, after delivery  **3.** Yes, before and after delivery | | | | | | | | | | | | | | | | | \|___\| | | | |
| 1. Admission or transfer to ICU?   0. No  1. Yes, before delivery  2. Yes, after delivery | | | | | | | | | | | | | | | | | \|___\| | | | |
| **Has any of the following clinical changes occurred during hospitalization:** | | | | | | | | | | | | | | | | | | | | |
| 1. Acute cyanosis?   0. No  1. Yes, before delivery  2. Yes, after delivery | | | | | | | | | | | | | | | | | | | \|___\| | |
| 1. Gasping?   0. No  1. Yes, before delivery  2. Yes, after delivery | | | | | | | | | | | | | | | | | | | \|___\| | |
| 1. Respiratory rate (RR)> 40 or <6 ipm?   0. No  1. Yes, before delivery  2. Yes, after delivery | | | | | | | | | | | | | | | | | | | \|___\| | |
| 1. Shock ?   0. No  1. Yes, before delivery  2. Yes, after delivery | | | | | | | | | | | | | | | | | | | \|___\| | |
| 1. Oliguria not responsive to fluids or diuretics?   0. No  1. Yes, before delivery  2. Yes, after delivery | | | | | | | | | | | | | | | | | | | \|___\| | |
| 1. Clotting failure?   0. No  1. Yes, before delivery  2. Yes, after delivery | | | | | | | | | | | | | | | | | | | \|___\| | |
| 1. Jaundice in the presence of preeclampsia?   0. No  1. Yes, before delivery  2. Yes, after delivery | | | | | | | | | | | | | | | | | | | \|___\| | |
| 1. Uncontrollable fit/total paralysis?   0. No  1. Yes, before delivery  2. Yes, after delivery | | | | | | | | | | | | | | | | | | | \|___\| | |
| 1. Stroke?   0. No  1. Yes, before delivery  2. Yes, after delivery | | | | | | | | | | | | | | | | | | | \|___\| | |
| 1. Loss of consciousness lasting ≥ 12 hours?   0. No  1. Yes, before delivery  2. Yes, after delivery | | | | | | | | | | | | | | | | | | | \|___\| | |
| 1. Loss of consciousness and absence of pulse/heartbeat?   0. No  1. Yes, before delivery  2. Yes, after delivery | | | | | | | | | | | | | | | | | | | \|___\| | |
| **The woman had any of the following laboratory abnormalities while in hospital:** | | | | | | | | | | | | | | | | | | | | |
| 1. Oxygen saturation <90% for ≥ 60 minutes   0. No  1. Yes, before delivery  2. Yes, after delivery | | | | | | | | | | | | | | | | | | | \|___\| | |
| 1. Pa O2/FiO2 <200 mmHg?   0. No  1. Yes, before delivery  2. Yes, after delivery | | | | | | | | | | | | | | | | | | | \|___\| | |
| 1. Creatinine ≥ 3.5 mg / dl?   0. No  1. Yes, before delivery  2. Yes, after delivery | | | | | | | | | | | | | | | | | | | \|___\| | |
| 1. Bilirubin> 6 mg / dl?   0. No  1. Yes, before delivery  2. Yes, after delivery | | | | | | | | | | | | | | | | | | | \|___\| | |
| 1. pH <7.1?   0. No  1. Yes, before delivery  2. Yes, after delivery | | | | | | | | | | | | | | | | | | | \|___\| | |
| 1. Lactate > 5?   0. No  1. Yes, before delivery  2. Yes, after delivery | | | | | | | | | | | | | | | | | | | \|___\| | |
| 1. Acute thrombocytopenia (platelets <50,000)?   0. No  1. Yes, before delivery  2. Yes, after delivery | | | | | | | | | | | | | | | | | | | \|___\| | |
| 1. Loss of consciousness and the presence of glucose and ketoacids in urine?   0. No  1. Yes, before delivery  2. Yes, after delivery | | | | | | | | | | | | | | | | | | | \|___\| | |
| **Did the woman receive any of the following treatments while in hospital:** | | | | | | | | | | | | | | | | | | | | |
| 1. Use of continuous vasoactive drugs (dopamine, dobutamine, epinephrine)?   0. No  1. Yes, before delivery  2. Yes, after delivery | | | | | | | | | | | | | | | | | | | \|___\| | |
| 1. Hysterectomy following infection, sepsis or hemorrhage?   0. No  1. Yes, before delivery  2. Yes, after delivery | | | | | | | | | | | | | | | | | | | \|___\| | |
| 1. Transfusion of ≥ 5 units of red blood cells?   0. No  1. Yes, before delivery  2. Yes, after delivery | | | | | | | | | | | | | | | | | | | \|___\| | |
| 1. Dialysis for acute renal failure?   0. No  1. Yes, before delivery  2. Yes, after delivery | | | | | | | | | | | | | | | | | | | \|___\| | |
| 1. Intubation and ventilation for ≥ 60 minutes not related to anesthesia?   0. No  1. Yes, before delivery  2. Yes, after delivery | | | | | | | | | | | | | | | | | | | \|___\| | |
| 1. Cardiopulmonary resuscitation?   0. No  1. Yes, before delivery  2. Yes, after delivery | | | | | | | | | | | | | | | | | | | \|___\| | |

**Attention! In the case of stillbirth, answer only the questions**

**172, 173, 174, 175, 176, 177, 178, 179, 180, 181, 216, 217, 218, 219, 251, 253, 254**

| **9. Newborn data – part 1** | | | | | | |
| --- | --- | --- | --- | --- | --- | --- |
| 1. Newborn medical record   \|___\|___\|___\|___\|___\|___\|___\|___\|___\|__\|___\|___\| | | | | | | |
| 1. Live Birth certificate registry number   1ª\|___\|___\|___\|___\|___\|___\|___\|___\|  2ª\|___\|___\|___\|___\|___\|___\|___\|___\| | | | | | | |
| 1. Sex: 1. Male   2. Female  3. Undefined | | | 1ª\|___\|  2ª \|___\| | | | |
| 1. Birthweight (grams): | | | 1ª\|___\|___\|___\|___\|g  2ª \|___\|___\|___\|___\|g | | | |
| 1. Length at birth (centimeters): | | | 1ª\|___\|___\|cm  2ª\|___\|___\|cm | | | |
| 1. Cephalic perimeter at birth: | | | 1ª\|___\|___\|cm  2ª\|___\|___\|cm | | | |
| 1. Gestational age by LMP | | | \|___\|___\| weeks   - 1. \|___\| days | | | |
| 1. Gestational age by ultrasound scan | | | \|___\|___\| weeks   - 1. \|___\| days | | | |
| 1. Gestational age by Capurro assessment or the New Ballard assessment | | | \|___\|___\| weeks   - 1. \|___\| days | | | |
| 1. Gestational age without specified method: | | | \|___\|___\|weeks   - 1. \|___\| days | | | |
| 1. Apgar score at 1 minute: | | 1ª\|___\|___\|  2ª \|___\|___\| | | | | |
| 1. Apgar score at 5 minutes: | | 1ª \|___\|___\|  2ª \|___\|___\| | | | | |
| **10. NEWBORN DATA - PART 2** | | | | | | |
| 1. Resuscitation in the delivery room 0. No 1. Yes | | | | | 1ª \|___\|  2ª \|___\| | |
| 1. O2 inhaled 0. No 1. Yes | | | | | 1ª \|___\|  2ª \|___\|  2ª \|___\|  3ª \|___\|  4ª \|___\| | |
| 1. Mask ventilation with an Ambu bag 0. No 1. Yes | | | | | 1ª \|___\|  2ª \|___\| | |
| 1. Orotracheal intubation 0. No 1. Yes | | | | | 1ª \|___\|  2ª \|___\| | |
| 1. Cardiac massage 0. No 1. Yes | | | | | 1ª \|___\|  2ª \|___\| | |
| 1. Drugs 0. No 1. Yes | | | | | 1ª \|___\|  2ª \|___\| | |
| 1. Other 0. No 1. Yes | | | | | 1ª \|___\|  2ª \|___\| | |
| 1. If other, specify __________________________________________________________________ | | | | | | |
| **Other procedures performed within the first hour after birth:** | | | | | | |
| 1. Upper airway aspiration? 0. No 1. Yes | | | | | | 1ª \|___\|  2ª \|___\| |
| 1. Gastric aspiration? 0. No 1. Yes | | | | | | 1ª \|___\|  2ª \|___\| |
| 1. Vitamin K (Kanakion)? 0. No 1. Yes | | | | | | 1ª \|___\|  2ª \|___\| |
| 1. Credé (drops of silver nitrate on newborn`s eyes)? 0. No 1. Yes | | | | | | 1ª \|___\|  2ª \|___\| |
| 1. Hepatitis B vaccination? 0. No 1. Yes | | | | | | 1ª \|___\|  2ª \|___\| |
| 1. Was the baby put in a baby incubator, nursery of heated crib after the delivery room?   0. No 1. Yes | | | | | | 1ª \|___\|  2ª \|___\| |
| 1. Was the baby admitted to a Neonatal Intermediate Care Unit:   0. No 1. Yes | | | | | | 1ª \|___\|  2ª \|___\| |
| 1. Was the baby admitted to a Neonatal Intensive Care Unit:   0. No 1. Yes | | | | | | 1ª \|___\|  2ª \|___\| |
| 1. The newborn needed to be transferred to a neonatal unit in another hospital   0. No 1. Yes | | | | | | 1ª \|___\|  2ª \|___\| |
| **Use of oxygen after birth:** | | | | | | |
| 1. Oxyhood 0. No 1. Yes | | | | 1ª \|___\|  2ª \|___\| | | |
| 1. Continuous positive airway pressure (CPAP) 0. No 1. Yes | | | | 1ª \|___\|  2ª \|___\| | | |
| 1. Mechanical ventilation 0. No 1. Yes | | | | 1ª \|___\|  2ª \|___\| | | |
| 1. Within 28 days of life was the baby on Oxygen therapy (any type)?   0. No 1. Yes 8. The baby was no longer in hospital | | | | 1ª \|___\|  2ª \|___\| | | |
| 1. If the baby was born premature, when he completed 36 weeks of corrected gestational age was he still on oxygen therapy (any type)?   1. No  2. Yes  3. Term newborn  4. Has not reached 36 weeks yet  5. The baby was no longer in hospital | | | | 1ª \|___\|  2ª \|___\| | | |
| 1. Use of surfactant: 0. No 1. Yes | | | | 1ª \|___\|  2ª \|___\| | | |
| 1. Hypothermia protocol performed 0. No 1. Yes | | | | 1ª \|___\|  2ª \|___\| | | |
| 1. Hypoglycemia (blood glucose less than 40) in the first 48 hours of life   0. No 1. Yes | | | | 1ª \|___\|  2ª \|___\| | | |
| 1. Minimum blood glucose in the first 48 hours of life | | | | \|___\|___\| | | |
| 1. Antibiotic use   1. No (go to question 211)  2. Before 48 hours of life (early sepsis) 3. After 48hours of life (late sepsis) | | | | 1ª \|___\|  2ª \|___\| | | |
| 1. How many days of antibiotic use? | | | | 1ª \|___\|___\|  2ª \|___\|___\| | | |
| 1. What antibiotics? __________________________________________________________________________________________________________________________________________________________________________________________________ | | | | | | |
| 210.1. What antibiotics? __________________________________________________________________________________________________________________________________________________________________________________________________ | | | | | | |
| 1. Blood culture? 2. No 1. Yes | | | | 1ª \|___\|  2ª \|___\| | | |
| 1. How many blood cultures? | | | | 1ª \|___\| \|___\|  2ª \|___\| \|___\| | | |
| 1. Phototherapy in the first 72 hours of life: 0. No 1. Yes | | | | 1ª \|___\|___\|  2ª \|___\|___\| | | |
| 1. Maximum level of bilirubin in the first 72 hours of life (mg / dl) | | | | 1ª\|___\|\|___\|,\|___\| mg/dl  2ª \|___\|\|___\|,\|___\| mg/dl | | |
| 1. Newborn blood type: 2. A 3. B 4. 0 5. AB 6. No registry | | | | 1ª \|___\|  2ª \|___\| | | |
| 1. Newborn Rh:   **0.** Negative  **1.** Positive  **9.** No registry | | | | 1ª \|___\|___\|  2ª \|___\|___\| | | |
| 1. Mother's blood type 2. A 3. B 4. 0 5. AB 6. No registry | | | | \|___\| | | |
| 1. Mother´s Rh:   **0.** Negative  **1.** Positive  **9.** No registry | | | | \|___\|___\| | | |
| 1. Congenital malformation?   0. Não  1. Sim | | | | 1ª \|___\|(If yes, answer question 220)  2ª \|___\|(If yes, answer question 220.a) | | |
| 1. Which? ­­­­­­­­­­­­­­­­­_____________________________________________________________________________________ | | | | | | |
| 220a Which? ­­­­­­­­­­­­­­­­­­­­­_____________________________________________________________________________________ | | | | | | |
| 1. Any surgery during hospitalization?   0. No  1. Yes | | | | 1ª \|___\| (If yes, answer question 222)  2ª \|___\| (If yes, answer question 222.a) | | |
| 1. Which? ___________________________________________________________________________________ | | | | | | |
| 222a. Which? ____________________________________________________________________________________ | | | | | | |
| **Other diagnoses during hospitalization:** | | | | | | |
| 1. Transient tachypnea? 0. No 1. Yes | | | 1ª \|___\|  2ª \|___\| | | | |
| 1. Hyaline membrane disease? 0. No 1. Yes | | | 1ª \|___\|  2ª \|___\| | | | |
| 1. Meconium aspiration syndrome? 0. No 1. Yes | | | 1ª \|___\|  2ª \|___\| | | | |
| 1. Pulmonary hypertension? 0. No 1. Yes | | | 1ª \|___\|  2ª \|___\| | | | |
| 1. Seizure? 0. No 1. Yes | | | 1ª \|___\|  2ª \|___\| | | | |
| 1. Necrotizing enterocolitis? 0. No 1. Yes | | | 1ª \|___\|  2ª \|___\| | | | |
| 1. Toxoplasmosis? 0. No 1. Yes | | | 1ª \|___\|  2ª \|___\| | | | |
| 1. Congenital rubella? 0. No 1. Yes | | | 1ª \|___\|  2ª \|___\| | | | |
| 1. Herpes? 0. No 1. Yes | | | 1ª \|___\|  2ª \|___\| | | | |
| 1. Cytomegalovirus? 0. No 1. Yes | | | 1ª \|___\|  2ª \|___\| | | | |
| 1. Congenital syphilis? 0. No 1. Yes | | | 1ª \|___\|  2ª \|___\| | | | |
| 1. Children exposed to HIV? 0. No 1. Yes | | | 1ª \|___\|  2ª \|___\| | | | |
| 1. Congenital Zika 0. No 1. Yes | | | 1ª \|___\|  2ª \|___\| | | | |
| 1. Other? | | | 1ª \|___\|  2ª \|___\| | | | |
| 236.1. Specify others here: | | | | | | |
| 1. Any birth injury?   0. No (go to question 242)  1. Yes | | | 1ª \|___\|  2ª \|___\| | | | |
| 1. Forceps or vacuum lesion 0. No 1. Yes | | | 1ª \|___\|  2ª \|___\| | | | |
| 1. Fractures (clavicle, femur, humerus or others)   0. No 1. Yes | | | 1ª \|___\|  2ª \|___\| | | | |
| 1. Other type of lesion:   **0.** No  **1.** Yes | | | 1ª \|___\| (If yes, answer 241)  2ª \|___\| (If yes, answer 241.a) | | | |
| 1. Quais? ______________________________________________________________________________________ | | | | | | |
| 241.a. Quais? _____________________________________________________________________________________ | | | | | | |
| 1. Exclusive breastfeeding:   **0.** No  1.Yes (go to question 244) | | | 1ª \|___\|  2ª \|___\| | | | |
| 1. Other foods that the baby received during hospitalization (allow more than 1 option)   1. Water 2. Intravenous glucose/ Oral glucose  3. human milk 4. Infant formula 5. Parenteral nutrition | | | 1ª \|___\|\|___\|\|___\|  2ª \|___\|\|___\|\|___\| | | | |
| 1. Type of hospital discharge   0. The baby was still in hospital after 28 days  1. Discharged from hospital  2. Neonatal death  3. Transferred to another hospital ( go to 246) | | | 1ª \|___\|  2ª \|___\| | | | |
| 1. Date of discharge/ death   (If neonatal death, go to question 251;  If hospital discharge, go to question 254) | 1ª\|___\|___\|/\|___\|___\|/\|___\|___\|___\|___\|  2ª \|___\|___\|/\|___\|___\|/\|___\|___\|___\|___\| | | | | | |
| 1. Hospital where the baby was transferred to (name - city - state)   ______________________________________________________________________________________________________________________________________________________________________________________________ | | | | | | |
| 1. Reason for being transferred   _______________________________________________________________________________________________ | | | | | | |
| 1. Date of transfer | 1ª \|___\|___\|/\|___\|___\|/\|___\|___\|___\|___\|  2ª \|___\|___\|/\|___\|___\|/\|___\|___\|\|___\|___\| | | | | | |
| 1. Type of discharge from hospital where the baby was transferred to: 2. The baby was still in hospital after 28 days (go to 254)   1. Hospital discharge  2. Neonatal death | | | | 1ª \|___\|  2ª \|___\| | | |
| 250. Date of hospital discharge/outcome from the hospital where he was transferred to: (If hospital discharge, go to question 254) | 1ª \|___\|___\|/\|___\|___\|/\|___\|___\|\|___\|___\|  2ª \|___\|___\|/\|___\|___\|/\|___\|___\|\|___\|___\| | | | | | |
| 251.Death cause registered in the newborn medical record   1. Extreme preterm baby (weight < 1000g) 2. Infection 3. Congenital Syphilis 4. Malformation 5. Respiratory complications 6. Others | | | | 1ª \|___\|\|___\|\|___\| (if others, answer question 252)  2ª \|___\|\|___\|\|___\|(if others, answer question 252.a) | | |
| 1. If other, specify here ______________________________________________________   _________________________________________________________________________________________________ | | | | | | |
| 252a. If other, specify here_________________________________________________________________________  _________________________________________________________________________________________________ | | | | | | |
| 1. Number of the death certificate registry   1ª\|___\|___\|___\|___\|___\|___\|___\|___\|  2ª \|___\|___\|___\|___\|___\|___\|___\|___\| | | | | | | |
| 1. Baby's weight (in grams) at hospital discharge, death or at 28 days after birth, if still hospitalized | | | 1ª \|___\|___\|___\|___\|g  2ª \|___\|___\|___\|___\|g | | | |
| 1. Comments: ___________________________________________________________________________________   _________________________________________________________________________________________________________________________________________________________________________________________________________________________________________________________________________________________________________________________________________________________________________________________________________________________________________________________________________________________________________________________________________________________________________________________________________________________________________________________________________________________ | | | | | | |
