# Supplementary material for: Evaluation of a quality improvement intervention for labour and birth care in Brazilian private hospitals: a protocol
Source: Reprod Health. 2018 Nov 26;15:194. doi: 10.1186/s12978-018-0636-y (PMC6257968; doi:10.1186/s12978-018-0636-y)
Supplement: Supplementary file 4 — Follow-up Telephone Interview Questionnaire. (DOCX 89 kb) [file 12978_2018_636_MOESM4_ESM.docx]

**
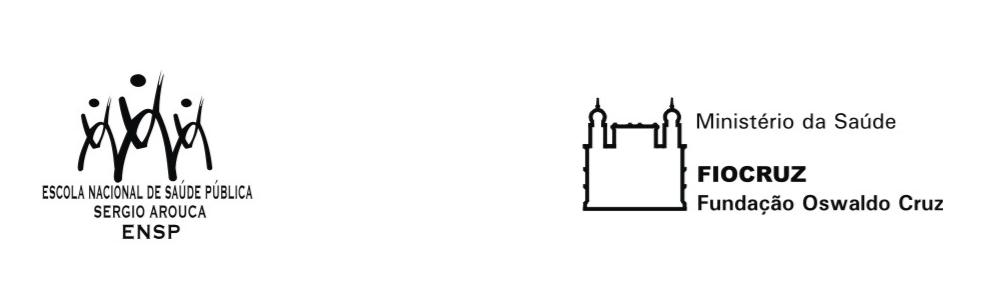
**

**TELEPHONE INTERVIEW - DATA COLLECTION INSTRUMENT**

RECORD ID |___|___|___|___|___|

| **1. GENERAL DATA** | | | | |
| --- | --- | --- | --- | --- |
| Hospital unit ____________________________________________________________________ | | | | |
| Name of post-partum woman _____________________________________________________________ | | | | |
| Name of interviewer ______________________________________________________________ | | | | |
| Today newborn age ________ days | | | | |
| Home phone number _____________________________________________________________ | | | | |
| Mobile phone number ____________________________________________________________ | | | | |
| Partner or relative phone number ____________________________________________________ | | | | |
| Partner or relative name ___________________________________________________________ | | | | |
| Work phone number _____________________________________________________________ | | | | |
| Birth date ____/____/_____ | | | | |
| Date of phone interview ____/____/_____ | | | | |
| Allowed phone contact 0. No 1. Yes | | | \|___\| | |
| Destination from hospital where the woman gave birth:  1. Discharged home/community from hospital  2. Transferred in the postpartum period  3. Left hospital without medical autorization  4. Death  5. Remained hospitalized after 42 days of birth | | | \|___\| | |
| Date of hospital discharge or death ____/____/_____ | | | | |
| Gestation type (single/twin) | | | | |
| Newborn name ________________________________________________________________________________ | | | | |
| Mode of delivery (vaginal/cesarean) | | | | |
| Newborn Condition (alive/dead) | | | | |
| Type of discharge from hospital where the baby was transferred to (rever)   1. The baby was still in hospital after 28 days (go to 254)   1. Hospital discharge  2. Neonatal death | | | | \|___\| |
| Date of newborn´s discharge or death ____/____/_____ | | | | |
| Phone call for woman or realative? | | ○ Yes  ○ No (it´s not possible after multiple calls)  ○ Waiting 43 days  ○ Trying | | |
| **INTERVIEW** | | | | |
| Good morning/afternoon, I am interviewer of a Fiocruz research team that is evaluating the birth and birth conditions in private Brazilian hospitals. You were recently interviewed by us during your hospital stay at the Hospital (name of the hospital). We're calling to find out how you and your baby are doing, and we'd like to know if you would agree to answer some questions, which will last around 5 minutes |  | ○ Agree (woman) (jump to question 2)  ○ Agree (respondent) (jump to question 1)  ○ Don´t agree (close interview) | | |
| 1. How is Mrs./Ms. _________________ (name) doing? |  | ○ Death (jump to question 1a)  ○ in Hospital (jump to question 3a)  ○ at home (jump to question 2) | | |
| A) What´s the date of _________________(name) death? |  | \|____\|____\|____\|  ○ Don´t know | | |
| B) What´s the cause of _______________ (name) death?    Jump to question 4 |  | ○ Hypertension / Preeclampsia / HELLP syndrome / Eclampsia  ○ Hemorrhage  ○ Infection  ○ Embolism  ○ Other? Which one?  ○ Don´t know | | |
| C) What´s the other reason for death? |  | ________________________ | | |
| 1. After your discharge, did you take antibiotics? |  | ○ Yes  ○ No (Jump to question 3)  ○ Don´t know | | |
| A) Why did you need to take antibiotics? |  | ○ operative wound infection  ○ mastitis  ○ episiotomy infection  ○ urinary infection  ○ other infections  ○ Don´t know | | |
| B) What´s the other infection? |  | ________________________ | | |
| 1. After your discharge from the hospital where you gave birth, have you been readmitted? |  | ○ Yes  ○ No (Jump to question 4)  ○ Don´t know | | |
| A) In which hospital were you readmitted? |  | ○ same hospital of birth  ○ different hospital. Which one? ___________________  ○ Don´t know | | |
| B) Date of readmission? |  | \|___\|___\|___\|  ○ Don´t know | | |
| C) How many days did the readmission last? |  | ______________ days  ○ Don´t know | | |
| D) What´s the reason for readmission? |  | ○ Hemorrhage  ○ Infection  ○ Embolism  ○ Hypertension  ○ Other? Which one?  ○ Don´t know | | |
| What´s the other reason for readmission? |  | ________________________ | | |
| 1. Have you been submitted to any surgery procedure during the readmission? |  | ○ Yes  ○ No (Jump to question 3g)  ○ Don´t know | | |
| 1. Have you been submitted to hysterectomy? |  | ○ Yes  ○ No  ○ Don´t know | | |
| G) Have you been submitted to blood transfusion during the readmission? |  | ○ Yes  ○ No  ○ Don´t know | | |
| 1. Have you been admitted in intensive care unit during the readmission? |  | ○ Yes  ○ No  ○ Don´t know | | |
| 1. How is _________________ (baby´s name) doing well? |  | ○ Yes (or any different answer of the two other options) (jump to question 5)  ○ Death  ○ Don´t know because he or she is living with other person (finish interview) | | |
| 1. When did ________________ (baby´s name) die? |  | \|___\|___\|___\|  ○ Don´t know | | |
| 1. What´s the death´s reason? |  | ○ Preterm consequences  ○ Respiratory reasons  ○ Cardiac reasons  ○ Infection  ○ Congenital malformations  ○ Diarrhea / Dehydratation  ○ Blood reasons  ○ Other cause  ○ Don´t know  **Finish interview** | | |
| What´s the other reason for death? |  | ________________________ | | |
| 1. After the discharge of hospital where you gave birth, have _________________ (baby´s name) been readmitted? |  | ○ Yes  ○ No (Finish interview)  ○ Don´t know (Finish interview) | | |
| A) In which hospital was he/she readmitted? |  | ○ same hospital of birth  ○ different hospital. Which one? ___________________  ○ Don´t know | | |
| B) What´s the date of readmission? |  | \|___\|___\|___\|  ○ Don´t know | | |
| C) How many days did the readmission last? |  | ______________ days  ○ Don´t know | | |
| D) What´s the reason for readmission? |  | ○ For phototherapy  ○ Low weight gain  ○ Dehydratation  ○ Infection  ○ Respiratory reasons  ○ Other  ○ Don´t know | | |
| What´s the other reason for readmission? |  | ________________________ | | |
| E) Have _____________ (baby´s name) been admitted in intensive care unit during the readmission? |  | ○ Yes  ○ No  ○ Don´t know | | |
| **Thanks for the interview and finish it.** | | | | |
