# Supplementary material for: Evaluation of a quality improvement intervention for labour and birth care in Brazilian private hospitals: a protocol
Source: Reprod Health. 2018 Nov 26;15:194. doi: 10.1186/s12978-018-0636-y (PMC6257968; doi:10.1186/s12978-018-0636-y)
Supplement: Supplementary file 5 — Script of Systematic Observation of the Hospital. (DOCX 40 kb) [file 12978_2018_636_MOESM5_ESM.docx]

**
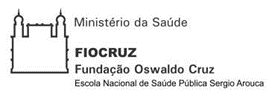

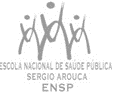
**

**SCRIPT OF A SEMI-STRUCTURED INTERVIEW**

(Key informants from the clinical staff - doctors and nurses - of the Adequate Childbirth Project in the hospital)

DATE OF THE INTERVIEW: ____/____/____

PERSON CONDUCTING THE INTERVIEW:___________________________________

**Objective:** To identify barriers and facilitating factors for the implementation of the Adequate Childbirth Project, from the adopters’ perspective.

Is the purpose of this interview clear? Then let us proceed.

**Identification**

1 – Name of the informant:_____________________________________________________________

2 – College degree:___________________________________________________________________

3 – Office held in the hospital:___________________________________________________________

4 – Your link to the Adequate Childbirth Project:_____________________________________________

**Decision making process and participation**

5 – How did the hospital decide to take part in the Adequate Childbirth Project? (In case the person is familiar with the hospital’s decision making process, ask how he or she was involved in it. And if the person isn’t familiar with it, ask what he or she was told about the hospital’s decision of taking part in the Adequate Childbirth Project).

6 – What is your perception of the Adequate Childbirth Project?

7 – Is your participation in the Adequate Childbirth Project mandatory or optional? What and/or who influenced your decision to take part in it? Which possible advantages and disadvantages do you identify, in taking part in the Adequate Childbirth Project?

8 – The hospital had to determine a target population and goals to be achieved by the end of the project. Are you aware of this target population and these goals? Could you describe them? Did you take part in the agreement with this target population and the goals? Do you agree with them? Why?

**Strategies**

9 – Where there any changes in the maternity ward thanks to the hospital’s participation in the Adequate Childbirth Project? Which ones?

10 – Did you have the opportunity of suggesting any changes?

11 – Which factors eased the implementation of these changes?

12 – Which factors hindered the implementation of these changes?

**Assistencial Practice**

13 – Did your daily work routine change after the start of the project? If so, how?

14 – Were there any changes in the way you assist laboring women, due to your participation in the project? In case yes, which changes?

(Let the professional speak freely, and in case he or she does not address the following topics, ask specifically about them:

# changes in the relationships with your colleagues who work in the obstetrical center – doctors and nurses;

# changes in the women’s care – interventions like episiotomy, oxytocin, Kristeller etc. and,

# changes in your criteria to indicate a C-section)? What or who was determinant for the changes in your professional practice (in case the person does not answer, ask if he or she took a training course sponsored by the Adequate Childbirth Project, or another entity.)

**Results**

15 – According to you, how do women, the hospital clients, react to the Adequate Childbirth Project?

16 – Have the results obtained by the Adequate Childbirth Project been reported to the staff? In which manner? Does the staff discuss these results?

17 – Do you know if the hospital natural childbirth rate has increased after the Adequate Childbirth Project? In your opinion, what is the reason for this increase, or non-increase?

18 – Do you notice any other results the hospital has gained because of the Adequate Childbirth project?

19 – Once the project is finished, do you believe that the changes will remain? Why?

20 – Is there any aspect of your participation in the Adequate Childbirth Project that wasn’t covered in this survey and that you would like to address?

Thank the respondent for the interview – Thank you very much for your participation!

Once all the managers of Adequate Childbirth Project are interviewed, the gathered data will be systematized and analyzed.
