# Supplementary material for: Evaluation of a quality improvement intervention for labour and birth care in Brazilian private hospitals: a protocol
Source: Reprod Health. 2018 Nov 26;15:194. doi: 10.1186/s12978-018-0636-y (PMC6257968; doi:10.1186/s12978-018-0636-y)
Supplement: Supplementary file 6 — Script of Qualitative Interview with Staff. (DOCX 41 kb) [file 12978_2018_636_MOESM6_ESM.docx]

**
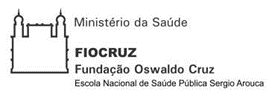

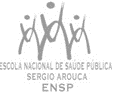
**

**SYSTEMATIC OBSERVATION SCRIPT**

**Objective**: To shed some light on the degree of re-invention within the interactions occurring in the care flow (among the leaders, frontline staff, as well as the pregnant and birthing women), and on the context of the ACP implementation.

**Observation Centers**

**Organization of the work process:** The aim is to learn about the division and sharing of tasks among all professionals involved in the women’s care (doctors, nurses, obstetrical nurses, nursing assistants), from admitting the woman, to assisting her labor, delivery and immediate postpartum. Notice the verbal and non-verbal communication processes – among the professionals – that express autonomy, responsibility, hierarchy and leadership.

**Trajectory of the care professional:** The aim is to understand a typical workday of the professional (doctor or obstetrical nurse), his or her role in assisting women to give birth (normal delivery or cesarean). Guiding questions: when and how does the professional approach the woman? How does the care relationship get established (interactions with the woman and her family, clinical decision-making process, sharing of cases with colleagues, time spent near or away from the care scene)? Describe the interactions in the places of exclusive access to professionals (medical staff and nursing staff, nurses station, coffee breaks, meeting room, etc.)? Observe the trajectory of the care professionals, particularly at the admission desk and at the obstetrical center, important places where they work and interact.

**Communication channels:** The aim is to observe and describe the strategies and results of the Adequate Childbirth Project for the women and professionals, in situations and places as: general meetings; rounds of the obstetrical center staff for the change of shift and day planning; murals and information panels – in accessible places and easy to spot for the staff and/or the users – posting the project’s goals as well as the results of the perinatal indicators; informal communication; informative banners or folders; quality reports of the patient’s safety nucleus, etc.

**Record of information:** The aim is to know how the data of the Adequate Childbirth Project is collected, including the profile of the person responsible for collecting it, the collection frequency, by which means (on paper or electronically), how this collection process is part of the workday and the importance assigned to it.

**Standards and protocols:** To observe the availability and use of the women’s care protocols, including her admission, labor, delivery and immediate postpartum. Guiding questions: Is there some physical material available (manuals)? Is it accessible? Is it being used? Are there algorithms, bundles, or some protocol abstract posted in places where the staff often gathers or walks by? What is described on paper is compatible with the staff’s conduct with the patients?

**Trajectory of the woman’s care:** Observe what happens to the woman, from her arrival in the hospital until her immediate postpartum. Guiding questions: How does she access the maternity service? Who welcomes her, how is she received? With whom does she interact most of the time? How does she express herself and how does the staff respond to her requests? How do they address her – “mammy,” “mom”? Do the professionals introduce themselves? Who speaks with her about giving birth? Who makes the decisions about her labor and delivery, and how? Is there room for the woman to express her needs about giving birth? Which elements pertaining to the woman’s trajectory in the maternity have an impact on her labor and delivery? Which elements unrelated to her care – services like manicure, makeup, filming, photography – are offered or requested by the woman during her trajectory in the maternity (does this occupy all of her time)? Is her privacy respected? Does she have a say in her own care? As you observe the care given to the woman, be attentive to how the interactions unfold.

**Environment:** To identify if the environment has elements that promote and foster normal childbirth. Guiding questions: Is there any information available to the women, about the advantages of normal childbirth? And about the Adequate Childbirth Project? Is there an incentive for the participation of a birth partner? Is there a place where the laboring women can walk? Do they use this place? Is it possible to control the lights, background noises and temperature? Are there non-pharmaceutical resources for pain relief? Are they used? Is it possible to guarantee the laboring woman’s privacy? Is privacy respected?

**To observe the professionals’ meetings**: The aim is to identify if they discuss in their meetings the goals of the Adequate Childbirth Project, as well as the results of indicators and improvement strategies, in addition to engaging in the discussion of clinical cases and scientific evidences.

**To observe a pregnancy group:** The aim is to identify if the group encourages normal childbirth and focuses mainly on women issues, whether there is room for the women to express their anxieties, share experiences and clarify doubts, or if the group meets in a class format, with little interaction among the women. This observation is aimed to identifying the topics discussed or reported as part of the discussion (when there is more than one meeting and the observation takes place only once), which method is used (participatory methodology, expositive lecture, etc), how the groups are scheduled (when and how often they take place, the existence of a closing activity), the conflicts that emerge during the meeting (whether linked to the topics being discussed or to the care flow).

**Places to observe**

(Focused on the characteristics of the maternity user’s flow)

- **Admission:** Reception room, examination room, ultrasound room; how are the women admitted; is there any information material, informative signs about relevant health related subjects and/or the guarantee of users’ rights? Is there any poster or informative flyer about the Adequate Childbirth Project?
- **Circulation between sectors:** Notice the women’s circulation flow in the maternity ward, check if it is limited or obstructed**.** How do they go from one sector to the other (wheelchair, walking)? Are they transferred from one sector to another while in labor?
- **Areas of circulation:** How do the women get around, and how are the places of care connected?
- **Nursing Station.**
- **Obstetrical Center**:
  1. Delivery rooms, notice how many they are and their use flow; does each have a bathroom?
  2. Equipment/materials that provide non-pharmaceutical pain relief (bobath ball, rocking chair, etc) are easy to spot and ready to be used? Do they have to be requested?
  3. Describe the rooms’ environment: their walls, decoration, furniture, how are the birthing devices laid out, and how do people move around them.
  4. Which equipment composes the environment (bed, lamp, chair, etc)?
- **Surgical birth environment.**
