# Supplementary material for: Evaluation of a quality improvement intervention for labour and birth care in Brazilian private hospitals: a protocol
Source: Reprod Health. 2018 Nov 26;15:194. doi: 10.1186/s12978-018-0636-y (PMC6257968; doi:10.1186/s12978-018-0636-y)
Supplement: Supplementary file 7 — Script of Qualitative Interview with Health Professionals. (DOCX 42 kb) [file 12978_2018_636_MOESM7_ESM.docx]

**
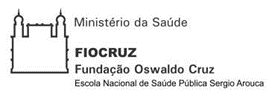

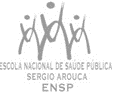
**

**SCRIPT OF A SEMI-STRUCTURED INTERVIEW**

(Members of the management staff responsible for implementing the Adequate Childbirth Project in the hospital – hospital director, Project leader in the hospital, head of obstetrics and head of the nursing staff of the obstetrical center)

DATE OF THE INTERVIEW: ____/____/____

PERSON CONDUCTING THE INTERVIEW:___________________________________

**Objective:** To identify barriers and facilitating factors for the implementation of the Adequate Childbirth Project. Is the purpose of this interview clear? Then let us proceed.

**Observation:** mention that you read the survey’s previous interview and now wish to further explore some of its aspects**.**

**Identification**

1 – Name of the respondent:_______________________________________________

2 – College degree:______________________________________________________

3 – Office held in the hospital:______________________________________________

4 – Role in the Adequate Childbirth Project:________________________________

**Decision-making process**

5 – What do you think of the hospital’s decision to take part in the Adequate Childbirth Project? How did the hospital learn about it? Whose decision was it to participate?

6 – What motivated the hospital to take part in the Adequate Childbirth Project?

7 – What motivated you to take on a leadership role in the Adequate Childbirth project?

8 – Prior to the do Adequate Childbirth Project was the hospital already developing other initiatives involving labor and delivery assistance? Which ones?

9 – Which possible advantages and disadvantages of taking part in the Adequate Childbirth project were identified?

10 – How was the decision to take part in the project communicated to the clinical staff? How did the staff react?

11 – Did you encounter any conflict and/or resistance to the Project’s implementation? Which ones? How did you counteract them?

12 – The hospital had to determine a target population and goals to be achieved by the end of the project. Describe the process of determining this target population and these goals? Did you take part in this process? How did the other members of the staff agree with the process? (Explore meeting minutes and notes.)

**Strategies**

13 – Which main strategies did the hospital adopt in order to reach the defined goals? Are you familiar with the directive diagram of the Adequate Childbirth Project (show it whichever the answer)? Would you say that these strategies were implanted?

14 – Who were your partners in defining and implementing the strategies?

15 – Which factors helped implementing these strategies?

16 – Which factors hindered the implementation of these strategies?

17 – Apart from the strategies proposed in the directive diagram of the Adequate Childbirth Project, did you develop any other specific strategies in the hospital? Which ones? Why did you decide to adopt these specific strategies?

**Participation of the health professionals**

18 – Which strategies have you been using in order to engage health professionals into taking part in the Adequate Childbirth Project?

19 – How would you qualify the doctors’ participation and involvement in the Adequate Childbirth project? And the nurses’ involvement?

**Participation of the women**

20 – Were the women, clients of the hospital, somewhat involved in defining and implementing the Adequate Childbirth Project’s strategies? How were they invited to participate? What kind involvement was it?

**Monitoring**

21 – How are the Project’s results registered? Who is responsible for registering them?

22 – Which other attributions are assigned to this professional?

23 – How does the staff learn about these results?

**Results of the strategy**

24 – Did the hospital’s normal childbirth rate increase after the Adequate Childbirth Project? To what do you attribute this result (increase or no-increase)?

25 – Would you highlight another result the hospital obtained from the Adequate Childbirth Project? Which one(s)?

26 – Once the Project is finished, do you believe that these changes will be maintained? Why?

27 – What did it mean to the hospital to take part in the Adequate Childbirth?

28 – Would you like to make an additional comment?

Thank the respondent for the interview – Thank you very much for your participation!

Once all the managers of the Adequate Childbirth Project are interviewed, the collected data will be systematized and analyzed.
