# Supplementary material for: Evaluation of a quality improvement intervention for labour and birth care in Brazilian private hospitals: a protocol
Source: Reprod Health. 2018 Nov 26;15:194. doi: 10.1186/s12978-018-0636-y (PMC6257968; doi:10.1186/s12978-018-0636-y)
Supplement: Supplementary file 8 — Script of Qualitative Interview with the Mothers. (DOCX 38 kb) [file 12978_2018_636_MOESM8_ESM.docx]

**
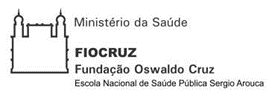

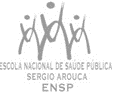
**

**SCRIPT OF A SEMI-STRUCTURED INTERVIEW**

(Postpartum mothers)

**Objective**: To identify the woman’s experience of the care she received and if she actively took part in the care flow.

Keep in mind that this interview is based on the guidelines of the woman-centered care, which aims at guaranteeing her autonomy and empowerment.

Guiding questions for the survey, directed to the women:

- Which was the women’s perception of the intervention suggested by the Adequate Childbirth Project?
- To what extent are the women’s experiences taken into consideration for the project’s improvement?
- Is the care flow of the theoretical model (directive diagram) compatible with what the women actually experience?

**Trigger question:** How was your birthing experience at the maternity X?

**Prenatal**

1 – How was your prenatal care? Where and with what professional?

2 – Did you attend a pregnancy group? Which themes were discussed in this group? Did you talk about labor and birth?

3 – How would you qualify the relationship with the professional? Did you feel welcome, well taken care of? What did you enjoy most in that relationship? Did something upset you? What?

4 – Information about pregnancy and birth: where/with whom did you get information; did a professional or a health insurance agent offer you information about pregnancy and birth? Did you share and/or get information about pregnancy and birth online? Where?

5 – During pregnancy, which were your expectations about giving birth? Did you devise a birth plan? Did you discuss your birth plan with the professional who saw you during your pregnancy?

6 – Did you share your expectations and/or information with friends and relatives?

7 – Did you use a prenatal card? If yes, proceed with the questions. Did you ask for the card or did a professional offer it to you? Do you know which kind of information it contained? In your opinion, which is the purpose of the prenatal card? What does the prenatal card mean to you? (In case she finds it difficult to answer these questions, ask again: Did the card help you be aware of how your pregnancy was unfolding? And making decisions?)

**Choice and visit to the maternity**

8 – How did you choose the maternity? Which characteristics did you consider important for your choice?

9 – Did you visit it? Why? If yes, what did you notice during the visit?

10 – During the visit, did they say anything about the Adequate Childbirth initiative? Do you know what it is?

**Childbirth: expectations x experience**

11 – What did you know about childbirth? Was this knowledge useful when you were giving birth? Which were your expectations about giving birth?

12 – Professional assistance during labor and delivery: who assisted you during labor and delivery? Was it the same professional who saw you at the prenatal visits? Do you think that the fact that it was (or not) the same person made a difference? Which pain relief resources where you offered?

13 – Labor companion: who accompanied you during your labor? How was this support?

14 – How was your experience of giving birth?

15 – Did you get to be in labor? In case yes, proceed: could you tell me about the interventions you received, while in labor? How were these interventions? Did you previously know about them? How did you experience these interventions?

**About the Adequate Childbirth strategy**

16 – Did a professional talk to you about the Adequate Childbirth initiative? How did you perceive this intervention proposed by the Adequate Childbirth Project? Were you asked if you had some changes to suggest in the proposal?

17 – When you arrived at the maternity, a doctor on shift assisted you, correct? How was the change from that doctor’s care to the next? Were you informed about this change? Did you notice any difference between their care?

18 – Would you say that within this initiative, your childbirth experience was taken into consideration for the project’s improvement? What should change?

19 – How did the Adequate Childbirth strategy help to achieve your expectations and honor your birth plan?

And if it didn’t, how could it have helped?

**Evaluation of childbirth**

20 – Now that a few months have gone by, how would you evaluate your childbirth experience?

**Directive Diagram Checklist**

21 – Is there compatibility between the care flow of the theoretical model and what the women experience?

22 – Devise a checklist for the women to assess the availability of the devices mentioned in the directive diagram.

Thank the respondent for the interview – Thank you very much for your participation!

Once all the interviews with the post-partum mothers are done, the collected data will be systematized and their content analyzed.
